# Association Between Genetically Proxied Lipid-Lowering Drug Targets and Renal Cell Carcinoma: A Mendelian Randomization Study

**DOI:** 10.3389/fnut.2021.755834

**Published:** 2021-10-12

**Authors:** Luyang Liu, Chao Sheng, Zhangyan Lyu, Hongji Dai, Kexin Chen

**Affiliations:** Department of Epidemiology and Biostatistics, National Clinical Research Center for Cancer, Key Laboratory of Molecular Cancer Epidemiology of Tianjin, Tianjin Medical University Cancer Institute and Hospital, Tianjin, China

**Keywords:** lipid-lowering drug, renal cell carcinoma, mendelian randomization, sex-specific, lipoprotein

## Abstract

Observational studies suggested inconsistent associations between lipid-lowering drugs, such as statins, and renal cell carcinoma (RCC) risk. In a two-sample Mendelian randomization (MR) framework, we assessed the causal influence of lipid-lowering agents and circulating lipid traits on overall and sex-specific RCC risk. Genetic variants of six drug-target genes were selected to proxy the effects of low-density lipoprotein cholesterol (LDL-C) lowering therapies. Instrumental variables for circulating lipid traits were constructed from two large genome-wide association studies. We used endpoints for RCC from summary statistics of two studies [International Agency for Research on Cancer [IARC], *N* = 13,230; National Cancer Institute [NCI], *N* = 4,735]. The robustness of results was assessed through conventional MR sensitivity analyses. Overall, there was no significant association between genetically proxied HMG-CoA reductase (HMGCR) inhibition and RCC risk [Odds ratio [OR] = 1.42, 95% CI, 0.29–6.99]. In the sex-stratified analysis, we observed a positive association for genetically proxied drug targets with RCC risk. Specifically, genetically proxied proprotein convertase subtilisin/kexin type 9 (PCSK9) inhibition was associated with a higher risk of RCC in men [OR = 2.20 [95% CI, 1.24–3.89]], and the difference by sex was moderate. This study suggested genetically proxied inhibition of HMGCR was not associated with RCC risk, while genetically proxied PCSK9 inhibition might be associated with a higher risk of RCC in male.

## Introduction

According to the estimates of cancer incidence and mortality reported by the International Agency for Research on Cancer (IARC), there were ~430,000 new cases diagnosed with renal cell carcinoma (RCC) and 180,000 deaths worldwide in 2020 (1). RCC patients are insensitive to conventional cytotoxic chemotherapy, cytokine therapy, and radiotherapy, and the underlying mechanisms are still unclear; moreover, currently, there is no efficient screening strategy for RCC (2). Therefore, primary prevention of RCC is necessary for reducing the disease burden.

Clear cell RCC (ccRCC) is characterized by the accumulation of lipid droplets in the cytoplasm. Both fatty acid synthesis and lipid storage could promote the growth of ccRCC (3). Observational studies also suggested that dyslipidemia might be involved in the carcinogenesis of RCC (4). However, a recent Mendelian randomization (MR) study that incorporated the largest published circulating lipid traits genome wide association study (GWAS) and RCC GWAS in European ancestry reported that there was no causal association for the influence of low-density lipoprotein cholesterol (LDL-C), high-density lipoprotein cholesterol (HDL-C), total cholesterol (TC), and triglyceride (TG) on RCC risk (5).

Statins are the inhibitors of 3-hydroxy-3-methylglutaryl coenzyme A (HMG-CoA) reductase and are the most commonly prescribed lipid-lowering agents that are widely used in both primary and secondary prevention of cardiovascular diseases (CAD). Recent researches have reported its cancer preventive effects, including promoting apoptosis, suppressing angiogenesis, and inhibiting tumor growth and metastasis (6). However, no increase of HMG-CoA reductase (*HMGCR*) activity was observed in RCC, making the role of statins in the prevention of RCC confused (7). In addition, growing epidemiological studies have investigated the association between statin use and the risk of RCC (8–11). However, the conclusions of these studies are controversial. Several studies reported a risk reduction of RCC in statin users (8, 9); while a nationwide case-control study indicated no chemopreventive effect of long-term use of statin on RCC (10), and another population-based study in Korea reported that statin elevated the risk of kidney cancer (11). Findings of traditional observational studies may be biased due to confounding factors, reverse causal association, and residual confounding, making the interpretation of these findings challenging. Some clinical trials have also explored the effect of lipid-modifying drugs on cancer risk; for example, a phase 3 clinical trial that was designed to assess the clinical efficacy and safety of anacetrapib [cholesteryl ester transfer protein [CETP] inhibitor] has reported a slightly increased but not significant risk of genitourinary cancer [relative risk [RR]: 1.08, 95% CI: 0.93–1.27] (12). However, due to limited follow-up periods and small numbers of RCC cases in clinical trials, it is difficult to make causal inferences for the relationship between lipid-lowering therapies and RCC risk.

Mendelian randomization is a method designed for causal inference using genetic variants to construct instrument variables. Genetic variants are randomly allocated at conception, and they are largely independent of potential confounders and reverse causality, which is common in conventional observational studies (13). Thus, MR may minimize confounding factors and provide more credible causal effect estimates without any potentially harmful interventions. With the rapid development of fundamental theory and growth of applications, drug-target MR analysis gradually becomes an efficient tool that can be applied to infer the influence of agents targeting protein-encoding genes, antagonists, agonists, activators, or inhibitors on disease risk (14). Compared to molecule-specific MR analysis, drug-target MR analysis generated instruments using genetic variants in DNA sequences located within or near genes, which encode the drug target to predict the effect of the corresponding drug. These variants may alter the expression or function of target genes (15). Yamolinsky et al. employed this method and identified a reverse relationship between genetically proxied HMGCR inhibitor and ovarian cancer risk (16). Using a similar study design, Luo et al. found a risk reduction effect of metformin on cardiovascular and cancer risk (17).

In this study, we performed drug-target and molecule-specific MR analyses to estimate the causal effects of circulating lipid traits and variants in genes encoding lipid-modifying drug targets on the risk of RCC under a two-sample MR framework. We aimed to evaluate whether genetically proxied lipid-lowering drugs and circulating lipid traits could influence the risk of RCC in both men and women.

## Materials and Methods

### Study Design

**Figure 1:**
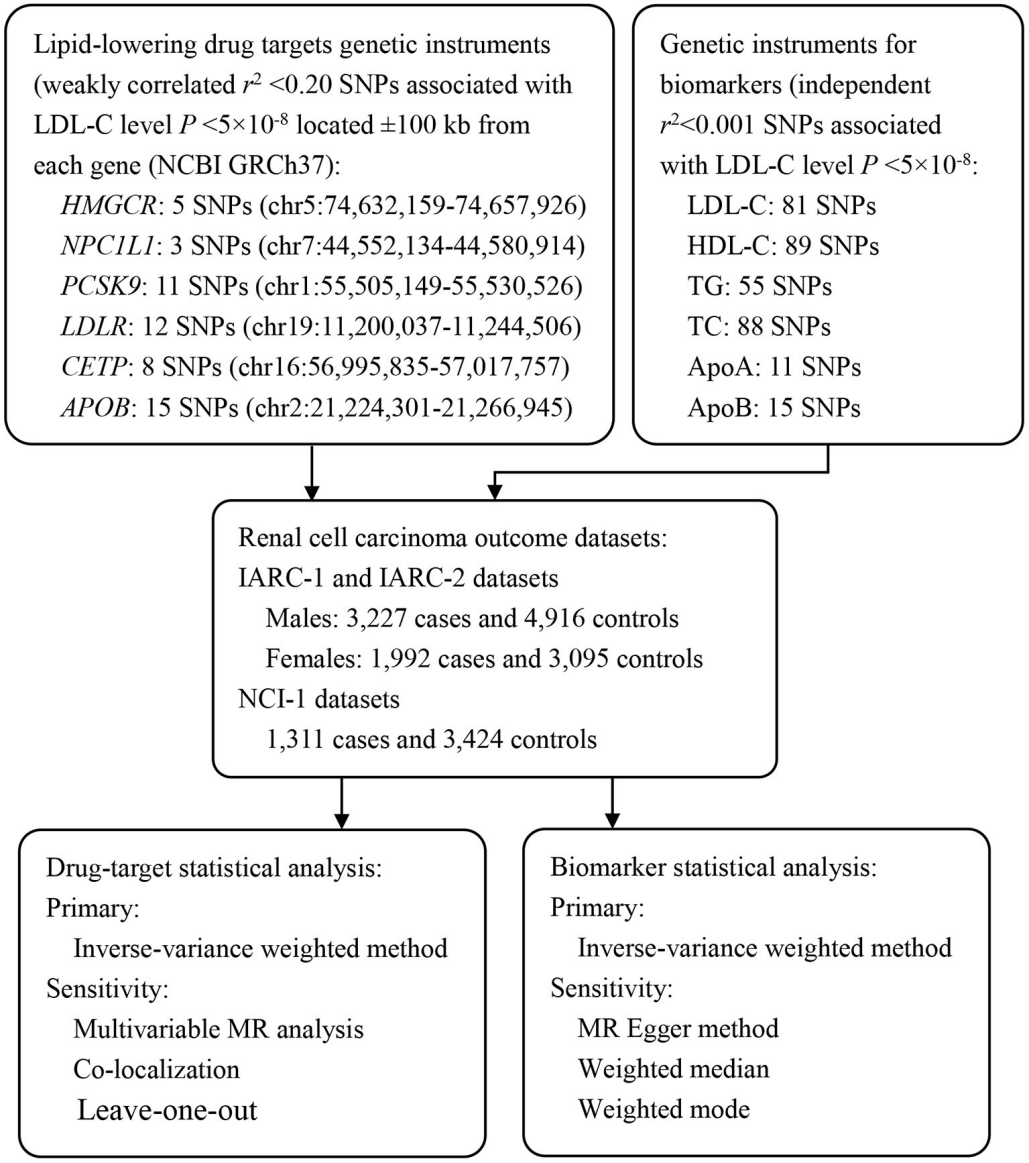
Overview of the study design. To construct instruments for drug targets and circulating biomarkers, summary genetic association data with LDL-C, HDL-C, TG, and TC were obtained from a European ancestry GWAS conducted by GLGC (*N* = 188,577). These SNPs were then matched with RCC outcome datasets to obtain SNP-outcome associations. After matching SNPs across traits and aligning them into the same effect allele, Mendelian randomization analyses were performed using the IVW method as the primary analyses, and various sensitivity analyses were applied to test Mendelian randomization assumptions (exchangeability and exclusion restriction). *HMGCR*, 3-Hydroxy-3-Methyl-Glutaryl-Coenzyme A Reductase; *NPC1L1*, Niemann-Pick C1-Like 1; *PCSK9*, Proprotein Convertase Subtilisin/Kexin type 9; *LDLR*, Low Density Lipoprotein Receptor; *CETP*, Cholesteryl Ester Transfer Protein; *APOB*, Apolipoprotein B; LDL-C, Low Density Lipoprotein Cholesterol; HDL-C, High Density Lipoprotein Cholesterol; TG, Triglyceride; TC, Total Cholesterol; ApoA, Apolipoprotein A; ApoB, Apolipoprotein B; MR, Mendelian Randomization; IARC, The International Agency for Research on Cancer; NCI, The National Cancer Institute.

We designed the main analysis and secondary analysis *in priori*. In the main analysis, we performed drug-target MR analysis to investigate the association of genetically proxied *HMGCR* (targets of statins), Niemann-Pick C1-Like 1 (*NPC1L1*, targets of ezetimibe), proprotein convertase subtilisin/kexin type 9 (*PCSK9*, target of evolocumab and alirocumab), *CETP* (target of anacetrapib), low density lipoprotein receptor (*LDLR*), and *APOB* (target of mipomersen) inhibitors with overall and sex-specific RCC risk. *LDLR* was not a specific drug target of any lipid-modifying agent, however, it was involved in the lipid metabolism, thus, we also assessed the role of the LDLR pathway in relation to the risk of RCC in our analysis. In the secondary analysis, we conducted a molecule-specific MR analysis to explore the causal relationship of circulating lipid traits, including LDL-C, HDL-C, TC, TG, ApoA, and ApoB, with overall and sex-specific RCC risk. Details of the study design were shown in Figure 1.

### Data Source

Data used in our study were publicly available from large-scale GWASs. All studies were approved by their respective institutional review board and were in concordance with the Declaration of Helsinki. Informed consent was also obtained from each participant.

To generate instrument variables for lipid-lowering drug targets, we obtained summary statistics from a GWAS of LDL-C conducted by the Global Lipid Genetics Consortium (GLGC) (18). Participants of European ancestry from 23 studies (*N* = 94,595) were genotyped with customized GWAS chips and individuals from 37 studies (*N* = 93,982) were genotyped using Metabochip arrays. In both studies, standard quality control and imputation to the 1,000 Genomes Project reference panel were performed. Circulating lipid levels of individuals treated without taking lipid-lowering drugs recently were measured after 8 h fasting. The Association test of each SNP was performed using linear regression with the inverse normal transformed trait values as the dependent variable and the allele count for each individual as the independent variable (18). To proxy drug-target effects, we applied a linkage disequilibrium (LD) clumping method. Specifically, we selected variants that were located within the ±100 kb range of each target gene and associated with LDL-C at a genome-wide significant threshold (*P* < 5 × 10^−8^). We then clumped these SNPs according to LD *r*^2^ ≤ 0.2 and a physical distance of 250 kb. For instruments with <3 SNPs, we further relaxed the LD *r*^2^ threshold to 0.40 to enlarge the variance explained by the instruments. Finally, there were 5 SNPs for proxy of *HMGCR*, 4 SNPs for *NPC1L1*, 11 SNPs for *PCSK9*, 12 SNPs for *LDLR*, 8 SNPs for *CETP*, and 15 SNPs for *APOB* (Table 1).

**Table 1:** Characteristics of LDL Cholesterol-Lowering Genetic Variants within/near *HMGCR, NPC1L1, PCSK9, LDLR, CETP*, and *APOB* gene.

**SNP**	**EA/NA**	**EAF**	**Effect (95% CI)^†^**	***P*** **-value**
**HMGCR**				
rs7711235	A/G	0.73	−0.038 (−0.050, −0.025)	5.00 × 10^−10^
rs3857388	T/C	0.87	−0.042 (−0.054, −0.031)	2.20 × 10^−11^
rs10515198	G/A	0.90	−0.060 (−0.072, −0.048)	5.99 × 10^−22^
rs12916	T/C	0.57	−0.073 (−0.081, −0.066)	7.79 × 10^−78^
rs12173076	T/G	0.88	−0.065 (−0.076, −0.054)	2.33 × 10^−27^
**NPC1L1**				
rs217386	A/G	0.81	−0.049 (−0.058, −0.039)	1.92 × 10^−21^
rs2073547	A/G	0.41	−0.036 (−0.044, −0.029)	1.20 × 10^−19^
rs17655652	C/T	0.29	−0.028 (−0.037, −0.019)	2.18 × 10^−10^
rs7791240	T/C	0.91	−0.043 (−0.055, −0.030)	1.84 × 10^−10^
**PCSK9**				
rs2479394	A/G	0.71	−0.039 (−0.047, −0.031)	1.58 × 10^−19^
rs11206510	C/T	0.15	−0.083 (−0.093, −0.073)	2.38 × 10^−53^
rs2479409	A/G	0.67	−0.064 (−0.072, −0.056)	2.52 × 10^−50^
rs11591147^*^	T/G	0.02	−0.497 (−0.532, −0.462)	8.58 × 10^−143^
rs11206514	C/A	0.39	−0.051 (−0.059, −0.043)	9.95 × 10^−33^
rs572512	C/T	0.65	−0.048 (−0.057, −0.039)	5.31 × 10^−26^
rs585131	C/T	0.18	−0.064 (−0.074, −0.054)	2.70 × 10^−35^
rs12067569^*^	G/A	0.97	−0.089 (−0.108, −0.069)	1.97 × 10^−17^
rs10493176	G/T	0.11	−0.078 (−0.098, −0.058)	2.54 × 10^−14^
rs11583974^*^	G/A	0.97	−0.065 (−0.088, −0.042)	3.95 × 10^−9^
rs2495477	C/T	NA	−0.064 (−0.075, −0.053)	7.29 × 10^−30^
**LDLR**				
rs12983316	A/G	0.83	−0.051 (−0.062, −0.041)	7.44 × 10^−22^
rs3786721	C/T	0.54	−0.047 (−0.054, −0.039)	2.89 × 10^−31^
rs12052058	T/G	0.25	−0.075 (−0.083, −0.067)	9.66 × 10^−62^
rs6511720	T/G	0.10	−0.221 (−0.233, −0.209)	1.00 × 10^−200^
rs73015030^*^	A/G	0.03	−0.152 (−0.181, −0.123)	2.62 × 10^−22^
rs1799898	T/C	0.15	−0.033 (−0.044, −0.023)	1.96 × 10^−9^
rs688	C/T	0.55	−0.054 (−0.061, −0.047)	1.01 × 10^−43^
rs2738464	G/C	0.13	−0.042 (−0.054, −0.030)	2.73 × 10^−10^
rs5742911	G/A	0.27	−0.061 (−0.072, −0.049)	4.83 × 10^−24^
rs892114	G/A	0.77	−0.035 (−0.045, −0.026)	7.63 × 10^−13^
rs7251031	T/G	0.71	−0.046 (−0.055, −0.037)	6.24 × 10^−23^
rs379309	T/C	0.50	−0.031 (−0.039, −0.024)	1.39 × 10^−13^
**CETP**				
rs12448528	G/A	0.77	−0.037 (−0.047, −0.027)	1.06 × 10^−12^
rs247616	T/C	0.29	−0.055 (−0.063, −0.047)	2.57 × 10^−37^
rs1864163	G/A	0.73	−0.044 (−0.053, −0.035)	7.97 × 10^−21^
rs9989419	G/A	0.59	−0.028 (−0.035, −0.020)	2.49 × 10^−12^
rs12920974	G/T	0.68	−0.032 (−0.043, −0.021)	2.96 × 10^−8^
rs9929488	G/C	0.70	−0.037 (−0.047, −0.028)	8.15 × 10^−13^
rs118146573	G/A	0.87	−0.053 (−0.069, −0.038)	1.02 × 10^−10^
rs289714	A/G	0.79	−0.036 (−0.046, −0.025)	2.85 × 10^−10^
**APOB**				
rs4665788	C/T	0.77	−0.067 (−0.075, −0.058)	1.12 × 10^−52^
rs11685356^*^	C/T	0.77	−0.052 (−0.060, −0.043)	1.21 × 10^−31^
rs6754295	G/T	0.26	−0.063 (−0.071, −0.055)	1.64 × 10^−47^
rs6725189	T/G	0.23	−0.060 (−0.069, −0.052)	5.63 × 10^−40^
rs533617^*^	C/T	0.05	−0.141 (−0.160, −0.121)	9.63 × 10^−45^
rs3791981	G/A	0.12	−0.094 (−0.107, −0.081)	2.03 × 10^−41^
rs12691202^*^	T/C	0.05	−0.097 (−0.119, −0.074)	8.22 × 10^−19^
rs12720842^*^	T/C	0.98	−0.099 (−0.122, −0.077)	1.88 × 10^−15^
rs12720796^*^	A/C	0.98	−0.091 (−0.119, −0.063)	1.68 × 10^−10^
rs1367117	G/A	0.71	−0.119 (−0.126, −0.111)	9.48 × 10^−183^
rs17398765	A/G	0.93	−0.092 (−0.107, −0.077)	3.54 × 10^−32^
rs7567653^*^	A/G	0.04	−0.115 (−0.136, −0.093)	3.37 × 10^−26^
rs515135	T/C	0.22	−0.139 (−0.149, −0.130)	1.09 × 10^−178^
rs6756743^*^	C/T	0.96	−0.055 (−0.073, −0.037)	4.97 × 10^−9^
rs113588790^*^	C/T	0.98	−0.090 (−0.118, −0.061)	3.94 × 10^−9^

* *These SNPs were not available in the RCC GWAS datasets, including three SNPs in PCSK9 (rs11591147, rs12067569, rs11583974), one SNP in LDLR (rs73015030), and seven SNPs in APOB (rs11685356, rs1291202, rs12720842, rs12720796, rs7567653, rs6756743, rs113588790)*.

† *Unit: 38.67mg/dL (1 SD)*.

*EAf, Effect Allele Frequency; HMGCR, 3-Hydroxy-3-Methyl-Glutaryl-Coenzyme A Reductase; NPC1L1, Niemann-Pick C1-Like 1; PCSK9, Proprotein Convertase Subtilisin/Kexin type 9; LDLR, Low Density Lipoprotein Receptor; CETP, Cholesteryl Ester Transfer Protein; APOB, Apolipoprotein B*.

To construct instruments for LDL-C, HDL-C, TG, and TC, we extracted variants associated with each lipid trait at a genome-wide significance (*P* < 5 × 10^−8^), LD *r*^2^ ≤ 0.001, and a physical distance ≥10 Mb from the GLGC GWAS summary data. There were 81, 89, 55, and 88 SNPs selected for LDL-C, HDL-C, TG, and TC instruments, respectively (Supplementary Table 2). Moreover, we included another two lipid traits, Apolipoprotein A (ApoA) and Apolipoprotein B (ApoB), from a recently published large-scale GWAS study that used nuclear magnetic resonance (NMR) metabolomics to quantify circulating metabolic traits with up to 24,924 European individuals (19). We used the same criteria and selected 11 and 15 SNPs for ApoA and ApoB, respectively (Supplementary Table 2).

The endpoints for RCC were selected from two RCC GWAS studies. All RCC cases were defined on the basis of the International Classification of Disease for Oncology, Second Edition (ICD-O-2) coded as C64 including all histological subtypes. And all controls were healthy participants recruited from large cohort studies. First, for the overall analysis, we used raw genotyping data obtained from dbGaP (phs000351.v1.p1), which recruited 1,311 cases and 3,424 controls of European ancestry conducted by the National Cancer Institute (NCI). We performed quality control, imputation, and association test as described in (20), and obtained association results of ~6.5 million SNPs. The data process procedure was presented in Supplementary Figure 1. In addition, we obtained summary genetic association statistics from a recently published sex-specific RCC GWAS (21). For sex-specific analysis, we obtained summary statistics from a publicly available dataset consisting of two IARC-Center National de Genotypage (CNG) scans with 5,219 RCC cases (1,992 women and 3,227 men) and 8,011 controls (3,095 women and 4,916 men) of European ancestry. IARC-2 study, the mean (SD) age of the participants was 60.16 (11.19) years. Quality control, imputation, and sex-specific association analyses protocols were described in a previous study (21). Finally, ~6.4 million SNPs were retained in the sex-specific GWAS dataset.

### Power Calculation and *F*-Statistic

Statistical power and *F*-statistics were calculated to ensure sufficient statistical power and avoid weak instrument bias. Power calculation was performed using online tools mRnd (http://cnsgenomics.com/shiny/mRnd) (22, 23). The statistical powers to capture an OR of 0.50 per one SD change in the circulating LDL-C levels were shown in Supplementary Table 3. The strength of each instrument was assessed by calculating *F*-statistics; typically, *F*-statistic >10 was considered to be no weak instrument bias (24).


$$\begin{array}{l} F\;=\;\frac{N\;-\;k\;-\;1}{k}\times \frac{R^{2}}{1\;-{\;R}^{2}} \end{array}$$

*N* indicated the sample size of the exposure factor, *k* indicated the number of SNPs in each instrument, and *R*^2^ represented the variance explained by the instrument. *R*^2^ was calculated according to the equation proposed by Shim et al. (25).

### Statistical Analysis

Data for SNP associations with LDL-C and with risk of RCC outcomes were harmonized to match coded effect alleles consistently. If the variants were not available for the outcomes, we searched for a proxy (*r*^2^ ≥ 0.8) for these SNPs. However, if no proxies were founded, the SNPs were omitted. Ambiguous SNPs with palindromic genotypes and minor allele frequencies between 0.4 and 0.5 were excluded from the analysis. We used the multiplicative random effect inverse-variance weighted (IVW) method to generate the overall estimate of causal effect when there are three or more variants in the instruments; while if there are two or fewer variants in the instruments, Wald ratio estimates were used. All the reported ORs of RCC risk were corresponding to one SD of LDL-C levels. In addition, for drug-target MR analysis, as there was weak LD (*r*^2^ ≤ 0.2) among the instruments, we included the correlation matrix of variants that were calculated based on the 1,000 Genomes Phase 3 reference panel for correction. We totally performed 18 tests and applied Benjamini-Hochberg false-discovery rate (FDR) procedure to adjust the raw *p-*values for multiple testing. All statistical tests were two-sided and a significant threshold was set to *P* < 0.05. We calculated the *p*-values for the sex disparity in effect estimates (based on log ORs and SEs for RCC). Specifically, we used a well-established formula to calculate the *z* statistics and then obtained the two-tailed *p*-values.


$$\begin{array}{l} z\;=\;\frac{\left(b_{1\;}-{\;b}_{2}\right)}{\sqrt{\left(SE_{b_{1}}^{2}\;+{\;SE}_{b_{2}}^{2}\right)}} \end{array}$$

where b_1_ and b_2_ are the MR effect estimates (log ORs and SEs for RCC), and SE_b1_ and SE_b2_ are the standard error of b_1_ and b_2_. Statistical analyses were conducted using the TwoSampleMR (v. 0.5.5), MVMR (v. 0.2.0), MendelianRandomization (v. 0.5.0), and MR-PRESSO (v. 1.0) packages in R (v. 3.5.3).

### Test of Basic MR Assumptions

Our study was based on a two-sample MR framework, which obtained SNP-exposure associations and SNP-outcome associations from diverse populations and sources to estimate the causal effects of exposure on the outcome (26). Therefore, three assumptions should be satisfied: (1) a strong link between genetic predictor and the exposure (“relevance”); (2) genetic predictor of the exposure is independent of the confounders influencing the relationship of exposure and outcome (“independence”); (3) genetic predictor affects the outcome only through the exposure of interest (“exclusion restriction”). We conducted three tests to test each assumption mentioned above. First, we applied adaption of *I*^2^-statistics (referred to as $I_{\text{GX}}^{2}$) to test whether there was no measurement error (i.e., NOME assumption) in the SNP-exposure association estimates. $I_{\text{GX}}^{2}$ statistics provided an estimation of the degree of expected relative bias (or dilution) in the MR-Egger causal estimate due to uncertainty in the SNP-exposure estimates (27). Simulation extrapolation (SIMEX) was then used to counteract the MR-Egger estimate for this dilution (28). Second, colocalization analysis was applied to test the independence assumption. Colocalization analysis investigated whether SNPs associated with both the exposures and outcomes were shared casual variants. This analysis was carried out with the eCAVIAR package and a threshold of colocalization posterior probability (CLPP) <0.01 was set to indicate a significant shared causal variant between drug-target instruments and RCC outcome (29). Third, multivariable MR analysis was applied to test the exclusion-restriction assumption. We included eight established risk factors (e.g., smoking, alcohol consumption; body mass index, BMI; waist-to-hip ratio; height; hypertension; diabetes; and chronic kidney disease) in the univariable MR model to assess the relationship between genetically proxied inhibition of drug targets and these RCC risk factors. We used multivariable MR analysis to adjust for statistically significant risk factors associated with genetically proxied inhibition of drug targets. Consistency of causal relationships between the adjusted and unadjusted model indicated a robust association that unlikely to be biased by other causal pathways (30). Extended descriptions of multivariable MR analyses were shown in Supplementary Methods.

### Sensitivity Analysis

Sensitivity analyses were performed using MR-Egger (31), weighted median (32), weighted mode, and MR-PRESSO (33) methods. Specifically, as there was weak LD (*r*^2^ ≤ 0.2) among instruments in the drug-target MR analysis, the residual genetic correlation was accounted for when applying these sensitivity analysis methods. MR-Egger intercept test was used to assess heterogeneity between causal effects of individual genetic variants. A pleiotropy test was applied to assess horizontal pleiotropy (34). In addition, we performed leave-one-out analyses to examine whether the removal of one SNP from the instruments iteratively influenced the overall estimates of a causal effect. To help validate the drug-target instrument construction strategies, we also used the same set of SNPs to assess the effect of LDL-C lowering on CAD. In this analysis (i.e., positive control analyses), we expected to see a significant causal effect of LDL-C lowering on CAD risk. Summary GWAS data on CAD risk were obtained from the CARDIoGRAM consortium (*N* = 22,233 cases, 64,762 controls) (35). All participants were European ancestry populations. Moreover, we also replicated the analysis using instrument variables constructed in previous MR studies (36–38). Information of SNPs to construct these genetic scores were listed in Supplementary Tables 13–17.

## Results

### MR Estimates

An overview of the study design was provided in Figure 1. Information of genetic variants in *HMGCR, NPC1L1, PCSK9, LDLR, CETP*, and *APOB* used to proxy the effect of drug-target genes were presented in Table 1 and Supplementary Table 1. Information of single nucleotide polymorphisms (SNPs), which used to generate instruments of LDL-C, HDL-C, TG, TC, ApoA, and ApoB were listed in Supplementary Table 2. Across the six drug-target instruments examined, *F*-statistics ranged from 64.2 to 369.6, indicating that weak instrument bias was unlikely to contribute to the analyses. Variance explained by the instruments ranged from 0.13 to 1.51%. For circulating lipid biomarkers analyses, the *F*-statistics of the instruments ranged from 68.1 to 153.6, and the explained variance ranged from 4.81 to 7.52% (Supplementary Table 3). The statistical power to capture an OR of 0.50 per one SD change in the circulating LDL-C levels in drug-target MR analyses was relatively low, ranging from 0.10 to 0.94.

**Figure 2:**
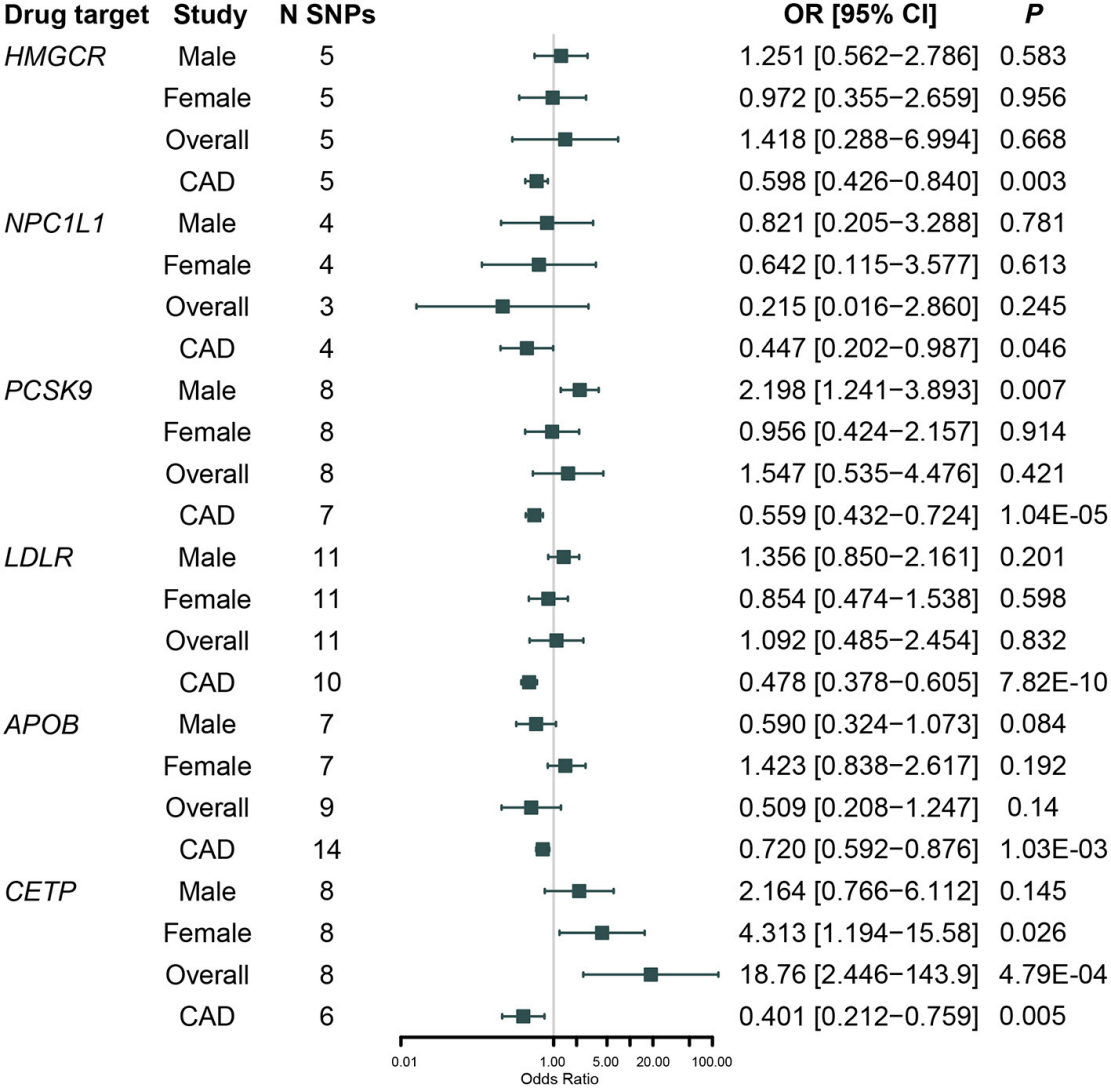
Association between genetically proxied inhibition of 3-Hydroxy-3-Methylglutaryl Coenzyme A Reductase (*HMGCR*), Niemann-Pick C1-Like 1 (NPC1L1), Proprotein Convertase Subtilisin/Kexin Type 9 (*PCSK9*), Cholesteryl Ester Transfer Protein (*CETP*), low-density lipoprotein receptor (*LDLR*), Apolipoprotein B (*APOB*) with renal cell carcinoma risk in NCI-1, IARC men and IARC women, and cardiovascular disease (CAD) risk in CARDIoGRAM after adjusted for weakly linkage disequilibrium (LD) among variants.

Results for the genetically predicted lipid-lowering drug targets on RCC and CAD risk adjusting for weak LD among variants were shown in Figure 2 and Supplementary Table 4. All these six drug targets were significantly associated with CAD risk (*P* < 0.05), indicating that the instruments were valid (Supplementary Table 4). We did not observe a significant association between genetically proxied HMGCR inhibition, which was equivalent to one SD reduction in LDL-C, and RCC risk [Odds ratio [OR] = 1.42, 95% CI, 0.29–6.99, *P* = 0.668]; and this association remained non-significant when classified by sex [men: OR = 1.25 [95% CI, 0.56–2.79], *P* = 0.583; women: OR = 0.97 [95% CI: 0.36–2.66], *P* = 0.956]. Similar results were observed in MR analyses for *NPC1L1* (*P* = 0.245), *LDLR* (*P* = 0.832), and *APOB* (*P* = 0.140) inhibitors.

We found that genetically proxied CETP inhibition was significantly associated with a higher risk of RCC [OR = 18.8 [95% CI, 2.45–143.9], *P* = 4.79 × 10^−4^, FDR-corrected *P* = 0.006]. Sex-specific analysis indicated that the association was nominally significant in women [OR = 4.31 [95% CI, 1.19–15.6], *P* = 0.026, FDR-corrected *P* = 0.112] rather than in men [OR = 2.16 [95% CI, 0.77–6.11], *P* = 0.145, FDR-corrected *P* = 0.305]; however, the difference by sex was not significant (*P*_difference_ = 0.41). In addition, although genetically proxied PCSK9 inhibition was not associated with RCC risk in the overall population [OR = 1.55 [95% CI, 0.54–4.48], *P* = 0.421, FDR-corrected *P* = 0.520], it was significantly associated with a higher risk of RCC in men [OR = 2.20 [95% CI, 1.24–3.89], *P* = 0.007, FDR-corrected *P* = 0.045] but not in women [OR = 0.96 [95% CI, 0.42–2.16], *P* = 0.914, FDR-corrected *P* = 0.701]; and the difference by sex was moderate (*P*_difference_ = 0.10).

**Figure 3:**
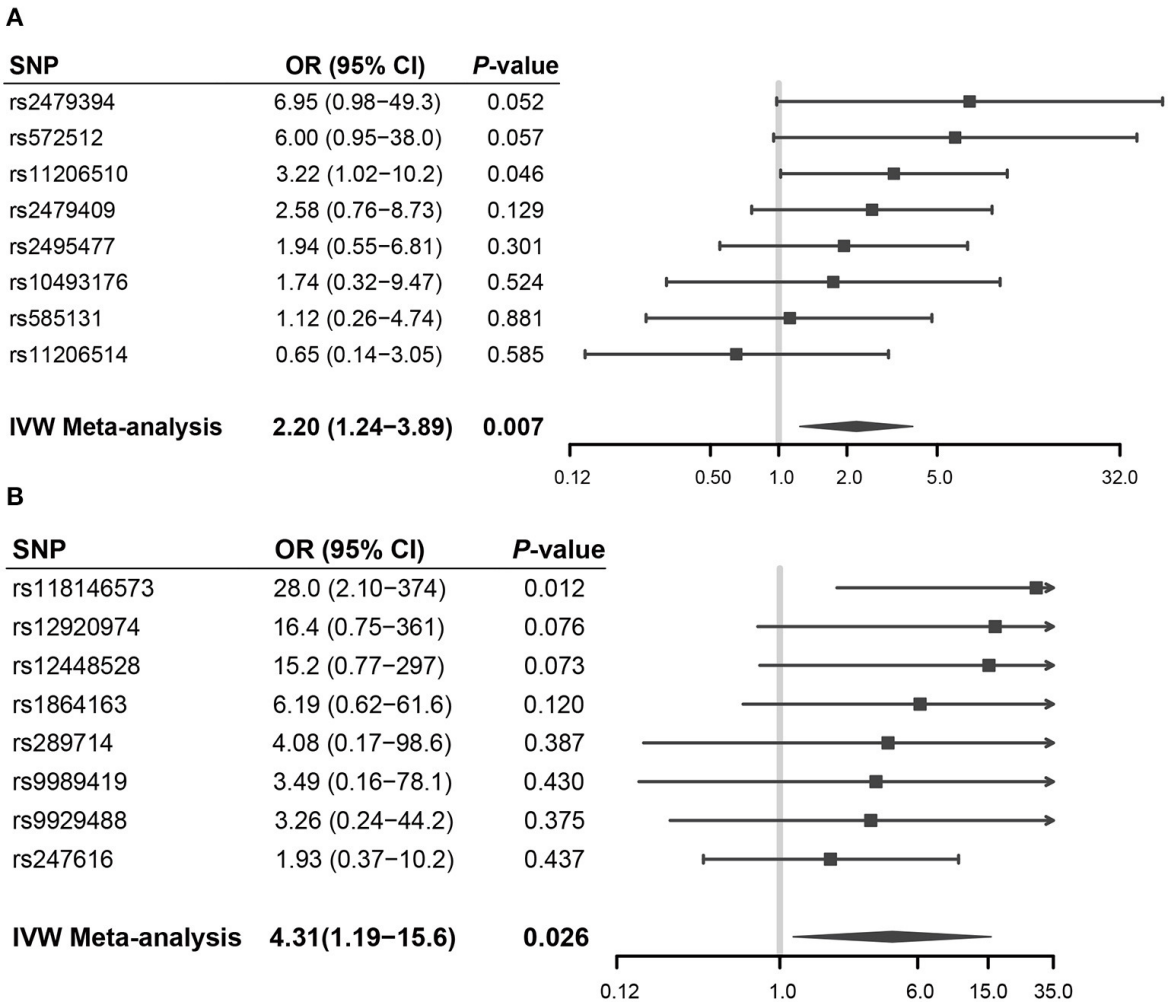
Forest plots showed the casual effect estimates of each SNP in PCSK9 and CETP inhibition instruments on RCC in men and women, respectively. **(A)** PCSK9 inhibitor and RCC risk in men; **(B)** CETP inhibitor and RCC risk in women.

Forest plots showed the casual effect estimates of each SNP in PCSK9 and CETP inhibition instruments on RCC in both men and women (Figure 3). Forest plots for other drug targets were shown in Supplementary Figure 2. We found that all the effect estimates of the SNPs in the *PCSK9* inhibition instrument on RCC risk in men were consistent except rs11206541. While all effect estimates of the SNPs in the *CETP* inhibition instrument on RCC risk in women were directionally consistent.

In the secondary analysis, we observed a nominally significant association between circulating TC level and RCC risk in men [OR = 1.20 [95% CI, 1.00–1.44], *P* = 0.044]. None of the other circulating traits was significantly associated with overall and sex-specific RCC risk (Supplementary Table 5).

### Basic MR Assumption Test Results

We found that none of the drug-target and lipid trait instruments was departed from the NOME assumption (all $I_{\text{GX}}^{2}$ >70%, Supplementary Table 6). We performed SIMEX-extrapolation MR-Egger tests to counteract the dilution and found consistent results with the standard MR-Egger method (Supplementary Table 7).

We observed no evidence that there were shared causal variants between LDL-C and RCC risk in men at *PCSK9* locus (rs11206510, CLPP = 0.004) and in women at CETP locus (rs247616, CLPP = 0.004) for a*-priori* defined CLPP threshold of 0.10. Detail results were shown in Supplementary Table 8.

Univariable MR analysis results of genetically proxied inhibition of drug targets and RCC risk factors were shown in Supplementary Table 9. We found that there was evidence for causal associations between *PCSK9* inhibitor instrument and lifetime smoking index [β = 0.02 [95% CI, 0.00, 0.03]; *P* = 0.028]; waist-to-hip ratio (β = 0.05 [95% CI, 0.03, 0.07]; *P* = 7.26 × 10^−6^]; type 2 diabetes risk [OR = 1.28 [95% CI, 1.09–1.51]; *P* = 0.002]. In addition, *CETP* inhibitor instrument was significantly associated with BMI [β = −0.06 [95% CI, −0.11, −0.01]; *P* = 0.017]; height [β = −0.06 [95% CI, −0.11, −0.01]; *P* = 0.025]; systolic pressure [β = −1.31 [95% CI, −2.19, −0.42]; *P* = 0.004]; and diastolic pressure [β = −0.59 [95% CI, −1.10, −0.074]; *P* = 0.025]. Results of multivariable MR analysis adjusted for those significant risk factors were shown in Supplementary Table 10. We found that casual associations of genetically proxied PCSK9 inhibition with RCC risk in men remained significant after adjusting for waist-to-hip ratio [OR = 1.59 [95% CI, 1.02–2.49], *P* = 0.041], lifetime smoking index [OR = 1.73 [95% CI, 1.02–2.93], *P* = 0.042], and type 2 diabetes [OR = 2.07 [95% CI, 1.33–3.24], *P* = 0.001]. However, the causal associations of genetically proxied CETP inhibition with RCC risk in women attenuated substantially after adjusting for BMI (*P* = 0.409), height (*P* = 0.647), systolic pressure (*P* = 0.985), and diastolic pressure (*P* = 0.765).

### Sensitivity Analysis

MR-Egger, weighted median, and weighted mode results were presented in Supplementary Tables 4, 5. For sensitivity analysis of drug-target MR, LD among SNPs was considered. We found that the effect estimates were consistent across these pleiotropy-robust methods.

**Figure 4:**
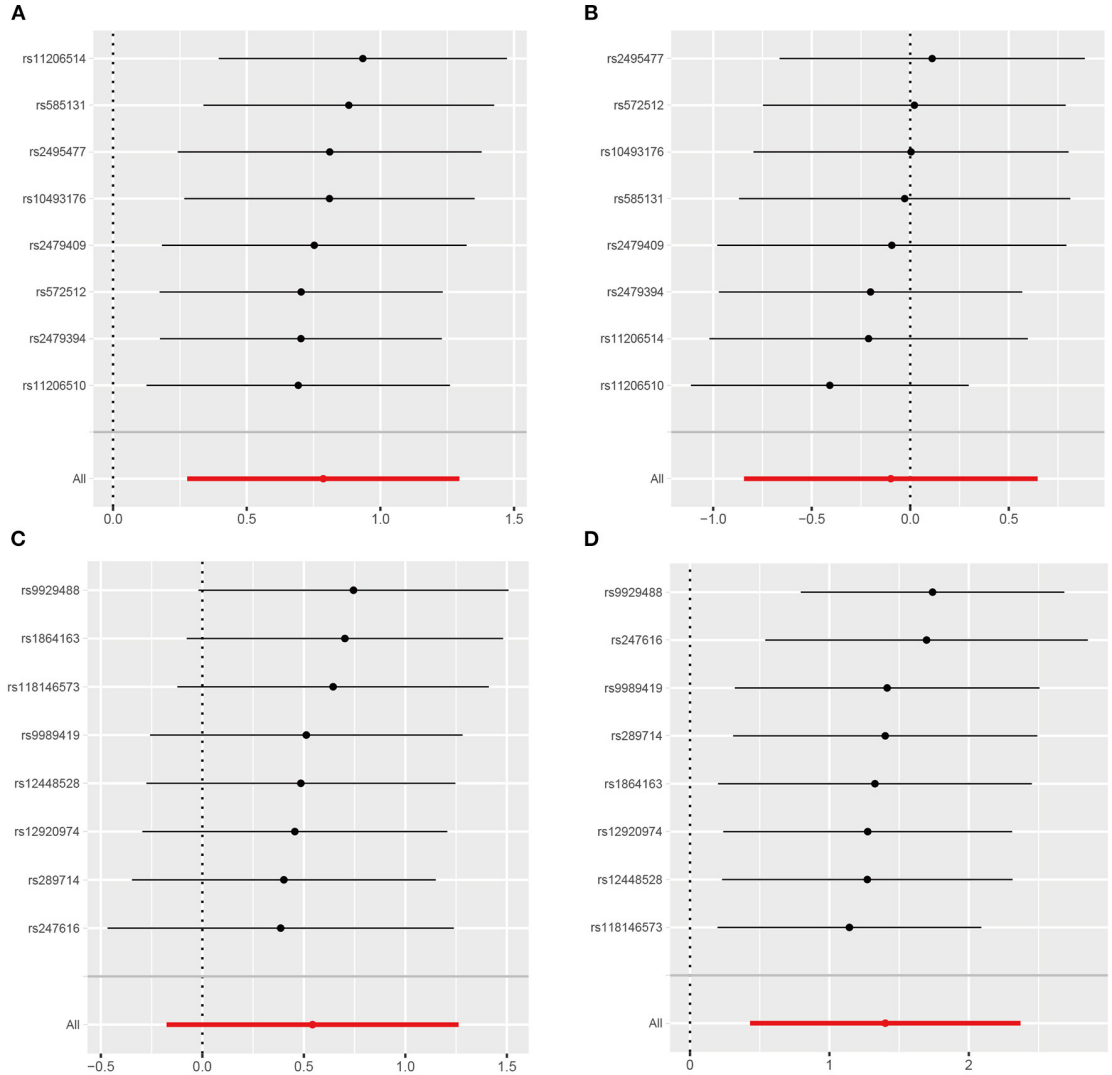
Leave-one-out analysis of genetically proxied PCSK9 and CETP inhibition on RCC risk in men and women. **(A)** PCSK9 inhibitor and RCC risk in men; **(B)** PCSK9 inhibitor and RCC risk in women; **(C)** CETP inhibitor and RCC risk in men; **(D)** CETP inhibitor and RCC risk in women.

Results of the leave-one-out analysis were shown in Figure 4 and Supplementary Figure 3. We found that causal effect estimates of genetically proxied inhibition of *PCSK9* with RCC risk in men and *CETP* with RCC risk in women were stable regardless of removal of any SNP in the instruments.

Heterogeneity and pleiotropy test results were presented in Supplementary Tables 11, 12. We observed some evidence for heterogeneity when assessing the causal effect of HDL-C and RCC risk in men (IVW *Q* = 115.95, *P* = 0.01; MR-Egger *Q* = 115.52, *P* = 0.01). Thus, we applied the multiplicative random-effect IVW meta-analysis method and found a consistent result [IVW OR = 1.20 [95% CI, 0.97–1.50], *P* = 0.093]. Pleiotropy tests showed no existence of significant horizontal pleiotropy (all *P* > 0.05).

In comparison analysis using lipid-lowering drug instruments previously reported, we also found that genetically proxied PCSK9 inhibition was causally associated with RCC risk in men [OR = 1.02 [95% CI, 1.00–1.04], *P* = 0.026] (Supplementary Table 18).

## Discussions

In this two-sample MR analysis involving 6,530 RCC cases and 11,435 controls of European ancestry, we found that genetically proxied long-term modulation of LDL-C levels by targeting *HMGCR* genes was not causally associated with reduced risk of RCC. However, genetically proxied PCSK9 inhibition was causally associated with a higher risk of RCC in men, but the difference by sex was not significant. In addition, very limited evidence was shown for an influence of circulating lipid traits, including LDL-C, HDL-C, TC, TG, ApoA, and ApoB, on the risk of RCC, which was consistent with the previous MR study.

### Association of HMGCR and RCC Risk

We observed no evidence of a protective effect of LDL-C lowering and genetically proxied inhibition of *HMGCR* on RCC risk. This result was directionally consistent with a recent MR study of 1,310 kidney cancer patients from UK Biobank, which found that genetically proxied inhibition of HMGCR was not associated with RCC risk [OR = 1.35 [95% CI, 0.51–3.57], *P* = 0.554] (39). Moreover, an umbrella review that reanalyzed the effect of statin on the risk of kidney cancer based on 4,052,120 participants from 11 studies has graded the evidence degree as non-significant (40). In addition, Gebhard et al. have reported that there was no increase in the HMGCR activity in RCC (7). However, it was worth noting that our findings were limited to the influence of LDL-C lowering effect of statins on RCC risk, and did not consider other effects of statins that could also influence the incidence of RCC. For example, it was reported that fluvastatin could potentiate the anticancer activity of vorinostat in renal cancer cells by activating the mechanistic target of rapamycin (mTOR) inhibitor (AMP)-activated protein kinase adenosine monophosphate (AMPK) (41). Therefore, further researches should focus on the potential mechanisms of statins on RCC risk beyond the LDL-C lowering effect.

### Association Between PCSK9 and RCC Risk

It is established that PCSK9 modulates lipid metabolism through degrading LDLR on the surface of hepatocytes (42). However, we found that genetically proxied LDLR inhibition was not causally associated with RCC risk. These results suggested that the mechanisms of PCSK9 inhibitors on RCC risk might be independent of LDL receptor degradation pathways. Previous genetic studies of PCSK9 inhibition suggested that reduced LDL-C levels by inhibiting *PCSK9* activity were significantly associated with a higher incidence of diabetes (43, 44). Hyperglycemia may contribute to the carcinogenesis of kidney cancer by dysregulation of the rennin-angiotensin system and AMP-activated protein kinase pathways (45, 46). In addition, *PCSK9* is expressed in the kidney, and involved in nephrogenesis; however, the function of PCSK9 in the kidney remains largely unknown (47). *In vivo* study indicated that PCSK9 interacted with epithelial sodium channel (ENaC) subunits and decreased their trafficking to the human embryonic kidney (HEK) 237 cell surface (48). Therefore, inhibiting PCSK9 might promote the trafficking of ENaC, while ENaCs are critically engaged in cancer cell biology, such as proliferation, migration, invasion, and apoptosis (49). Unfortunately, due to the lower prevalence and distinct pathological characteristics of RCC, and the shorter duration of the PCSK9 inhibitor trials, there were few epidemiology studies focused on the association of PCSK9 inhibitors and RCC risk. Further pharmacological researches uncovering the influence of PCSK9 inhibitors on the physiological function of renal cells are warranted. More genetic and observational studies are needed to elucidate the associations.

Interestingly, we found that genetically proxied PCSK9 inhibitor was only significantly associated with RCC risk in men rather than women, and the difference by sex was moderate. Sex disparity of PCSK9 levels has long been discussed. A clinical trial (NCT00848276) explored the relationship among testosterone, estradiol, and circulating PCSK9 levels and suggested that circulating PCSK9 was not related to or affected by testosterone in men, whereas inversely related to estradiol in women (50). Schooling CM et al. have investigated whether statins and PCSK9 inhibitors have pleiotropic effects on ischemic heart disease (IHD) *via* testosterone in men and women. And they demonstrated genetically proxied effects of statins other than PCSK9 inhibitors in men affected testosterone, which partly mediated effects of IHD (51). It is established that men are at substantially higher lifelong risk of RCC than women (~2-fold). Therefore, it should be more cautious for men with a higher risk of RCC when using PCSK9 inhibitor therapies.

### Strength and Limitations

To our knowledge, our study was the largest drug-target MR analysis so far to explore the causal effect of genetically proxied lipid-modifying agents on overall and sex-specific RCC risk. We comprehensively examined targets of commonly prescribed lipid-lowering drugs, including statins, ezetimibe, evolocumab, anacetrapib, and mipomersen, and a series of sensitivity analyses were applied to test whether the results violated basic assumptions or were biased by horizontal pleiotropy.

However, there were several limitations. The first limitation was the relatively small sample size for RCC risk GWAS studies, which resulted in low statistical power and a wide CI. However, participants in these studies were European ancestry populations recruited from European countries, Australia, and the USA, and they were well-representatives. In the future, larger RCC GWASs and more powerful instruments might enable us to estimate the causal effects more precisely. Second, selection bias might affect MR estimates when samples selected into the study are strongly influenced by the risk factor. However, restricted to the small sample size and insufficient information of the samples, selection bias cannot be ruled out in our study. Future studies with a larger sample size and novel statistical analysis methods (such as inverse probability weighting method) might result in more accurate effect estimates. Third, we could not use sex-specific instrumental variables for circulating lipid traits due to limited robust associations between SNP and the traits. However, a recently published sex-specific GWAS across 33 quantitative biomarker traits in UK Biobank demonstrated that sex played a limited role in the genetics of most traits (52). Thus, this bias should have little consequence for the result interpretation. The fourth limitation was that drug-target MR analysis was designed to reflect the effect of life-long modulation of lipid-lowering agents to modify LDL-C levels on the disease. It was unable to indicate the effect of short-term administration of lipid-modifying agents. Fifth, all GWAS samples were of mostly European ancestry, making extrapolation of our conclusions to other populations difficult. Sixth, given that the incidence rate of RCC increases steadily with age, with a peak of incidence at ~75 years, competing risk factors before recruitment might bias the results, which was so-called “winner's curse bias” (53). Seventh, this analysis considered no interaction of the association between genetic variants proxied to the drug targets and RCC risks, such as gene-environment interaction and gene-gene interaction. Eighth, our analysis only revealed the on-target effect of drug use solely and ignored the off-target consequences of related medication. Specifically, we only considered the causal influence of LDL-C lowering effect of lipid-modifying agents on RCC risk; however, the pharmacological mechanisms underlying each medication are more complex. For example, statin use has been reported to lead to extensive lipoprotein and fatty acid changes beyond LDL-C (54). Ninth, some instrument variables were not available in the RCC datasets, which might lead to the missing of the causal effect estimates. However, as these SNPs were in weak LD with each other and leave-one-out analyses were stable regardless of removal of any SNP in the instrument, the missing variants may not change the results substantially.

In conclusion, we found very limited evidence to support the genetically proxied inhibition of HMGCR as a causal protective factor for RCC. However, our results tentatively suggested that genetically proxied inhibition of *PCSK9* and *CETP* were significantly associated with a higher risk of RCC in a sex-specific manner. These findings provided insights into the potential mechanisms of action of the novel lipid-lowering therapies. However, as the pharmacological mechanisms of these medications were complex, we could not conclude that short-term administration of PCSK9 and CETP inhibitor therapies would increase RCC risk.

## Data Availability Statement

Summary statistics for circulating lipid traits produced by GLGC are available at: http://csg.sph.umich.edu/willer/public/lipids2013/. All meta-analysis results of circulating metabolic traits quantified by NMR are available through URL: http://www.computationalmedicine.fi/data/NMR_GWAS/. Genotype data of NCI-1 scan are accessible on dbGaP: phs000351.v1.p1. Genome-wide sex-specific summary statistics of IARC scan are publicly available through the NHGRI-EBI GWAS Catalog: https://www.ebi.ac.uk/gwas/downloads/summary-statistics.

## Author Contributions

All authors listed have made a substantial, direct and intellectual contribution to the work, and approved it for publication.

## Funding

This work was supported by the Chinese National Key Research and Development Project (Grant No. 2018YFC1315600) and Tianjin Municipal Education Commission (2016YD21).

## Conflict of Interest

The authors declare that the research was conducted in the absence of any commercial or financial relationships that could be construed as a potential conflict of interest.

## Publisher's Note

All claims expressed in this article are solely those of the authors and do not necessarily represent those of their affiliated organizations, or those of the publisher, the editors and the reviewers. Any product that may be evaluated in this article, or claim that may be made by its manufacturer, is not guaranteed or endorsed by the publisher.
