# Supplementary material for: Association Between Genetically Proxied Lipid-Lowering Drug Targets and Renal Cell Carcinoma: A Mendelian Randomization Study
Source: Front Nutr. 2021 Oct 12;8:755834. doi: 10.3389/fnut.2021.755834 (PMC8545796; doi:10.3389/fnut.2021.755834)
Supplement: Supplementary file 1 [file Data_Sheet_1.PDF]

Supplementary Online Content

Supplementary Methods

Supplementary Figure 1 Overview of data process in RCC GWAS conducted by NCI-1. All the quality control, imputation and association test process were performed according to protocols provided by previously published article.

Supplementary Table 1 Comparison of effect allele frequencies across GWAS for measured LDL-C levels, renal cell carcinoma in males and females.

Supplementary Table 2 Characteristics of genetic variants in LDL-C, HDL-C, TG, TC, ApoA and ApoB instruments.

Supplementary Table 3 *F*-statistics estimates for genetic instruments, variance explained by genetic instruments and statistical power (%) estimates for primary analyses.

Supplementary Table 4 Association of Genetically Proxied Inhibition of *HMGCR*, *NPC1L1*, *PCSK9*, *CETP*, *LDLR*, *APOB* with Overall and Sex-specific Renal Cell Carcinoma Risk after adjusted for weakly linkage disequilibrium (r^2 ≤0.20) among variants.

Supplementary Table 5 Mendelian randomization results of circulating lipid traits with overall and sex-specific renal cell carcinoma risk.

Supplementary Table 6 I^2_{GX} statistics to assess violation of the "NO Measurement Error" (NOME) assumption for instruments used in MR-Egger regression.

Supplementary Table 7 SIMEX corrected MR-Egger estimates.

Supplementary Table 8 Colocalization analysis of circulating LDL-C levels and risk of overall and sex-specific renal cell carcinoma for *PCSK9* and *CETP* variants.

Supplementary Table 9 Univariable Mendelian randomization analyses assessed the association between genetically-proxied inhibition of *HMGCR*, *NPC1L1*, *PCSK9*, *CETP*, *LDLR*, *APOB* and previously reported risk factors for renal cell carcinoma.

Supplementary Table 10 Association between genetically-proxied inhibition of *PCSK9*, *CETP* and renal cell carcinoma in males and females , adjusted for significant RCC risk factors identified by univariable MR analyses.

Supplementary Table 11 Assessing heterogeneity of single nucleotide polymorphism

(SNP) effect estimates in inverse-variance weighted (IVW) and MR-Egger regression.

Supplementary Table 12 Assessing directional pleiotropy through MR-Egger intercept.

Supplementary Figure 2 Drug-target Mendelian randomization analysis single SNP plots.

Supplementary Figure 3 Leave-one-out analysis plots.

Supplementary Table 13 *HMGCR* variants included in *HMGCR* genetic score and their association with LDL-C in the Global Lipids Genetics Consortium.

Supplementary Table 14 *NPC1L1* variants included in *NPC1L1* genetic score and their association with LDL-C in the Global Lipids Genetics Consortium.

Supplementary Table 15 *PCSK9* variants included in *PCSK9* genetic score and their association with LDL-C in the Global Lipids Genetics Consortium.

Supplementary Table 16 *LDLR* variants included in *LDLR* genetic score and their association with LDL-C in the Global Lipids Genetics Consortium.

Supplementary Table 17 *CETP* variants included in *CETP* genetic score and their association with LDL-C in the Global Lipids Genetics Consortium.

Supplementary Table 18 Association of Genetically Proxied Inhibition of *HMGCR*, *NPC1L1*, *PCSK9*, *CETP*, *LDLR*, *APOB* with Overall and Sex-specific Renal Cell Carcinoma Risk using instrument variables reported in previous articles.

Reference

Supplementary Methods

Multivariable Mendelian Randomization

We systematically searched for potential confounding factors which might influence plasma LDL-C levels and renal cell carcinoma (RCC) risk simultaneously. Finally, 6 potential confounding factors were involved in the analyses, including smoking, drinking, anthropometric traits, hypertension, diabetes and chronic kidney disease (CKD). For smoking, we included comprehensive smoking index (1) (a continuous variable indicating the smoking heaviness; *N* =462,690) and smoking initiation (2) (a categorical variable indicating whether the participants smoked regularly; *N* =1,232,091) GWAS conducted by GSCAN. For alcohol consumption, we extracted SNPs from drinks per week (*N* =941,280) GWAS conducted by GSCAN (1). For anthropometric traits, summary statistics of height, body mass index (BMI) (3) and waist to hip ratio (WHR) (4) were from GWAS meta-analysis of GIANT consortium and UK Biobank which consisted of ~0.7 million European populations. For blood pressure, we included systolic pressure and diastolic pressure GWAS meta-analysis conducted by ICBP and UK Biobank (*N* =757,601) (5). For diabetes, we included fasting glucose (*N* =46,186), fasting insulin (*N* =38,238) GWAS conducted by MAGIC consortium (6, 7) and type 2 diabetes fine-mapping study (*N* =149,821) conducted by DIAGRAM consortium (8). For CKD, we included large-scale GWAS recently conducted by CKDGen consortium (*N* =117,165), CKD patients were defined as estimated glomerular filtration rate (eGFR) <60mL/min/1.73m^2 (9). Univariable MR analyses were performed to assess the causal associations between genetically-proxied lipid-lowering drugs and these factors. For continuous outcome, we used β and 95% CI to indicate the alterations of the outcome corresponding to 1 SD of LDL-C levels; while for categorical outcome, we calculated OR and 95% CI to suggest the risk of outcome event corresponding to 1 SD decrease of LDL-C levels. For risk factors that were associated with drug targets (P <0.05), we further integrated GWAS summary statistics and additional instrument variables and conducted multivariable MR analyses to estimate the direct effect of lipid-lowering drugs independent of these confounding factors. Both IVW and MR-Egger method were

extended to estimate the causal effects in multivariable MR framework (10, 11). Generalized Cochran's *Q* tests were applied to assess instrument strength and validity. There were no overlapping samples for LDL-C and these confounding factors GWAS studies, thus the covariance was fixed at zero. Consistency of associations between analyses with and without adjustment for these confounding factors would suggest that analyses were unlikely to be biased by horizontal pleiotropy.

Comparison Analysis

For comparison, we repeated the analysis using variants for lipid-lowering drug instruments that have been used previously (12-15). Details were shown in previous studies. Briefly, for *HMGCR, NPC1L1*, *PCSK9, LDLR* and *CETP* genetic scores, using linear regression model to test the associations between variants within a 500 kb window of each gene and plasma LDL-C levels. All variants that were associated with LDL-C at a nominally significance (P <0.05) conditional on all other variants and in low linkage disequilibrium (r^2 <0.3) with each other were included (12-14).

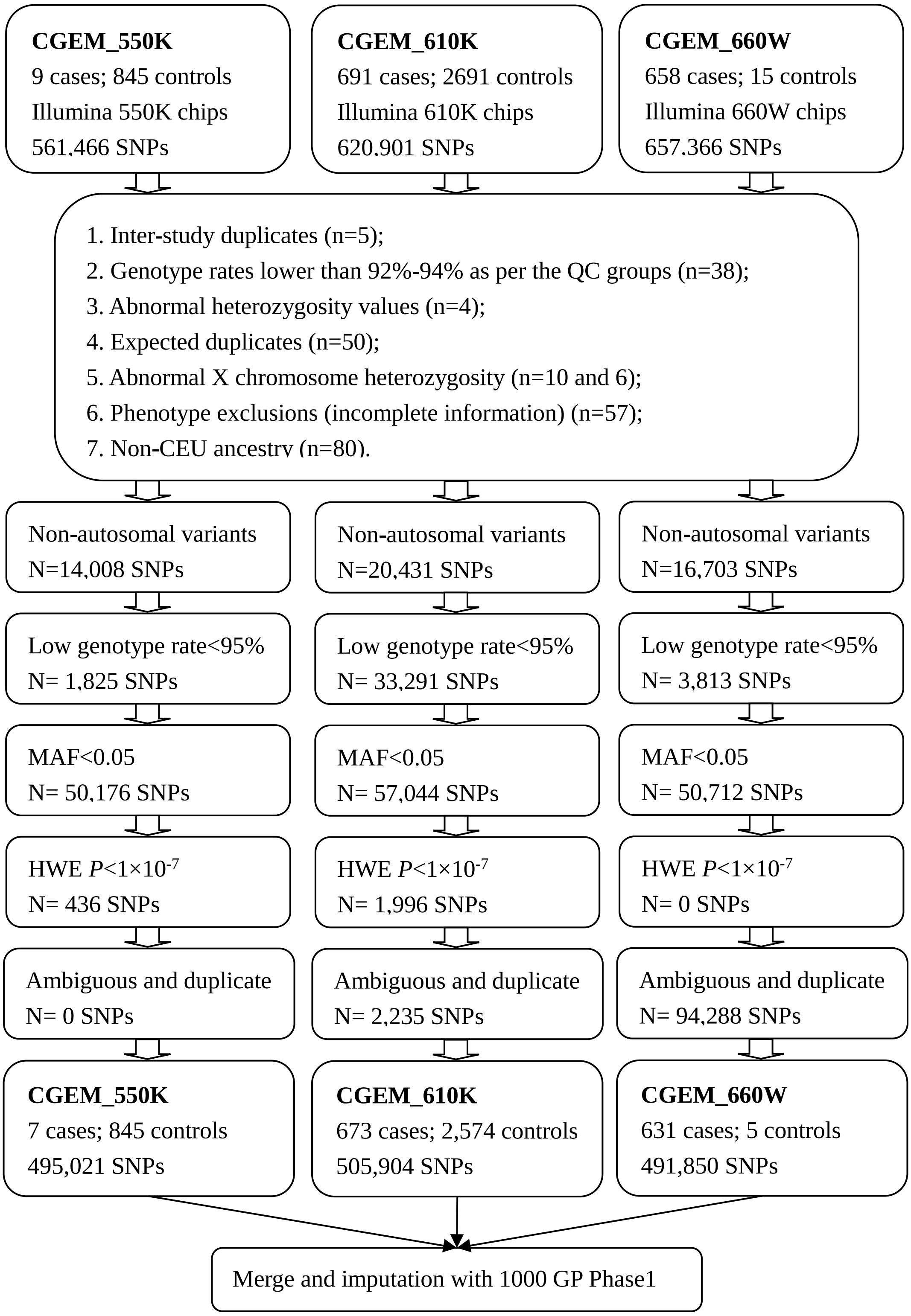

Supplementary Figure 1 Overview of data process in RCC GWAS conducted by NCI-1. All the quality control, imputation and association test process were performed according to protocols provided by previously published article.

Supplementary Table 1 Comparison of effect allele frequencies across GWAS for measured LDL-C levels, renal cell carcinoma in males and females**.**

SNP	LDL-C	NCI-1	IARC Male	IARC Female
HMGCR inhibition				
rs7711235	0.73	0.77	0.77	0.77
rs3857388	0.87	0.89	0.89	0.88
rs10515198	0.90	0.90	0.88	0.89
rs12916	0.57	0.57	0.58	0.58
rs12173076	0.88	0.88	0.89	0.89
NPC1L1 Inhibition				
rs217386	0.41	0.40	0.43	0.42
rs2073547	0.81	0.77	0.79	0.78
rs7791240	0.91	0.90	0.90	0.90
rs17655652	0.29	0.29	0.30	0.30
PCSK9 Inhibition				
rs2479394	0.71	0.72	0.72	0.72
rs11206510	0.15	0.18	0.17	0.17
rs2479409	0.67	0.67	0.65	0.66
rs11591147	0.02	NA	NA	NA
rs11206514	0.39	0.35	0.39	0.38
rs572512	0.65	0.64	0.65	0.66
rs585131	0.18	0.17	0.17	0.16
rs12067569	0.97	NA	NA	NA
rs10493176	0.11	0.13	0.10	0.10
rs11583974	0.97	NA	NA	NA
rs2495477	NA	0.39	0.40	0.41
LDLR Inhibition				
rs12983316	0.83	0.83	0.85	0.84
rs3786721	0.54	0.55	0.55	0.53

rs12052058	0.25	0.23	0.25	0.23
rs6511720	0.10	0.10	0.10	0.10
rs73015030	0.03	NA	NA	NA
rs1799898	0.15	0.13	0.14	0.13
rs688	0.55	0.55	0.59	0.59
rs2738464	0.13	0.12	0.11	0.12
rs5742911	0.27	0.30	0.32	0.32
rs892114	0.77	0.76	0.78	0.78
rs7251031	0.71	0.71	0.68	0.69
rs379309	0.50	0.52	0.51	0.51
CETP Inhibition				
rs12448528	0.77	0.77	0.77	0.77
rs247616	0.29	0.31	0.31	0.32
rs1864163	0.73	0.76	0.75	0.74
rs9989419	0.59	0.60	0.61	0.60
rs12920974	0.68	0.70	0.71	0.71
rs9929488	0.70	0.73	0.73	0.72
rs118146573	0.87	0.88	0.88	0.88
rs289714	0.79	0.82	0.82	0.81
APOB Inhibition				
rs4665788	0.77	0.75	0.75	0.74
rs11685356	0.77	0.77	NA	NA
rs6754295	0.26	0.27	0.23	0.23
rs6725189	0.23	0.21	0.19	0.21
rs533617	0.05	0.05	NA	NA
rs3791981	0.12	0.10	0.11	0.10
rs12691202	0.05	NA	NA	NA
rs12720842	0.98	NA	NA	NA
rs12720796	0.98	NA	NA	NA

rs1367117	0.71	0.71	0.68	0.68
rs17398765	0.93	0.94	0.93	0.93
rs7567653	0.04	NA	NA	NA
rs515135	0.22	0.19	0.18	0.17
rs6756743	0.96	NA	NA	NA
rs113588790	0.98	NA	NA	NA

Supplementary Table 2 Characteristics of genetic variants in LDL-C, HDL-C, TG, TC, ApoA and ApoB instruments.

SNP	EA/NEA	EAF	Effect (SE)	*P* value
**LDL-C**				
rs7534572	C/G	0.31	-0.0407,0.0058	1.29×10^{-11}
rs2642438	A/G	0.25	-0.0352,0.0042	7.32×10^{-16}
rs2587534	G/A	0.47	-0.0391,0.0037	8.06×10^{-25}
rs12066643	T/C	0.12	-0.0389,0.0064	1.06×10^{-8}
rs4970712	A/C	0.19	-0.0339,0.0044	2.46×10^{-13}
rs10903129	A/G	0.46	-0.0328,0.0037	3.03×10^{-17}
rs11591147	T/G	0.02	-0.4970,0.0180	8.60×10^{-143}
rs6693893	C/T	0.04	-0.0767,0.0132	2.88×10^{-8}
rs12748152	C/T	0.93	-0.0499,0.0066	3.21×10^{-12}
rs2495495	C/T	0.87	-0.0342,0.0059	3.52×10^{-8}
rs7551981	G/T	0.40	-0.0472,0.0038	1.36×10^{-33}
rs4970834	T/C	0.19	-0.1503,0.0047	1.00×10^{-200}
rs267733	G/A	0.14	-0.0331,0.0053	5.29×10^{-9}
rs72902576	G/T	0.04	-0.0933,0.0133	9.58×10^{-12}
rs2030746	C/T	0.60	-0.0214,0.0038	8.60×10^{-9}
rs16831243	C/T	0.82	-0.0378,0.0055	9.06×10^{-12}
rs1250229	T/C	0.21	-0.0243,0.0042	3.13×10^{-8}
rs1367117	G/A	0.71	-0.1186,0.0040	9.50×10^{-183}
rs6544713	C/T	0.71	-0.0806,0.0041	4.84×10^{-83}
rs6709904	G/A	0.11	-0.0550,0.0085	4.58×10^{-10}
rs2710642	G/A	0.38	-0.0239,0.0038	6.09×10^{-9}
rs10490626	A/G	0.08	-0.0508,0.0069	1.70×10^{-12}
rs10195252	C/T	0.42	-0.0238,0.0039	3.81×10^{-8}
rs11563251	C/T	0.87	-0.0345,0.0062	4.50×10^{-8}
rs17404153	T/G	0.14	-0.0336,0.0054	1.83×10^{-9}

rs7640978	T/C	0.11	-0.0392,0.0069	9.84×10^{-9}
rs9875338	A/G	0.39	-0.0270,0.0037	2.21×10^{-11}
rs6818397	G/T	0.59	-0.0224,0.0040	1.68×10^{-8}
rs12916	T/C	0.57	-0.0733,0.0038	7.79×10^{-78}
rs4530754	G/A	0.42	-0.0275,0.0036	3.58×10^{-12}
rs6882076	T/C	0.33	-0.0456,0.0038	3.31×10^{-31}
rs10947332	G/A	0.87	-0.0504,0.0056	6.97×10^{-18}
rs6909746	T/C	0.39	-0.0263,0.0037	7.86×10^{-11}
rs3757354	T/C	0.21	-0.0382,0.0044	2.09×10^{-17}
rs1408272	G/T	0.05	-0.0520,0.0083	3.68×10^{-9}
rs112201728	C/T	0.94	-0.0675,0.0104	8.51×10^{-10}
rs1564348	T/C	0.85	-0.0481,0.0050	2.76×10^{-21}
rs13206249	A/G	0.22	-0.0378,0.0062	4.53×10^{-8}
rs16891156	A/C	0.98	-0.0965,0.0171	8.23×10^{-9}
rs2315065	C/A	0.91	-0.1102,0.0158	5.23×10^{-12}
rs2390536	G/A	0.63	-0.0223,0.0038	2.04×10^{-8}
rs4722551	T/C	0.83	-0.0391,0.0049	3.95×10^{-14}
rs2073547	A/G	0.81	-0.0485,0.0049	1.92×10^{-21}
rs2737252	A/G	0.26	-0.0314,0.0041	7.04×10^{-14}
rs7832643	G/T	0.59	-0.0339,0.0038	2.67×10^{-17}
rs13277801	T/C	0.65	-0.0338,0.0038	3.99×10^{-17}
rs9987289	A/G	0.08	-0.0714,0.0066	8.53×10^{-24}
rs2954029	T/A	0.47	-0.0564,0.0036	2.10×10^{-50}
rs3780181	G/A	0.05	-0.0445,0.0074	1.76×10^{-9}
rs1883025	T/C	0.24	-0.0296,0.0044	6.14×10^{-11}
rs579459	T/C	0.78	-0.0665,0.0045	2.42×10^{-44}
rs2419604	G/A	0.68	-0.0302,0.0040	7.49×10^{-14}
rs174583	T/C	0.37	-0.0522,0.0038	7.00×10^{-41}
rs10893499	G/A	0.86	-0.0521,0.0053	3.86×10^{-21}

rs10832962	C/T	0.28	-0.0320,0.0040	6.62×10^{-14}
rs964184	C/G	NA	-0.0855,0.0078	2.01×10^{-26}
rs3184504	T/C	0.47	-0.0268,0.0038	4.20×10^{-12}
rs1169288	A/C	0.67	-0.0375,0.0040	6.45×10^{-21}
rs4942486	C/T	0.54	-0.0243,0.0037	2.26×10^{-11}
rs8017377	G/A	0.54	-0.0303,0.0038	2.52×10^{-15}
rs247616	T/C	0.29	-0.0547,0.0041	2.57×10^{-37}
rs2000999	G/A	0.82	-0.0650,0.0046	4.22×10^{-41}
rs1801689	A/C	0.96	-0.1028,0.0139	9.81×10^{-12}
rs314253	C/T	0.34	-0.0242,0.0038	3.44×10^{-10}
rs2886232	C/T	0.88	-0.0451,0.0064	3.88×10^{-11}
rs6504872	C/T	0.53	-0.0274,0.0037	3.48×10^{-13}
rs2228603	T/C	0.07	-0.1040,0.0072	4.43×10^{-44}
rs6511720	T/G	0.10	-0.2209,0.0061	1.00×10^{-200}
rs2965157	C/T	0.02	-0.1886,0.0112	7.29×10^{-62}
rs7254892	A/G	0.03	-0.4853,0.0119	1.00×10^{-200}
rs676388	T/C	0.54	-0.0265,0.0039	1.31×10^{-11}
rs2738459	C/A	0.44	-0.0532,0.0058	2.26×10^{-19}
rs75687619	G/T	0.98	-0.1735,0.0161	8.05×10^{-24}
rs12721109	A/G	0.02	-0.4462,0.0183	3.00×10^{-122}
rs2328223	A/C	0.75	-0.0299,0.0050	5.63×10^{-9}
rs6065311	T/C	0.54	-0.0417,0.0036	1.66×10^{-30}
rs1800961	T/C	0.03	-0.0685,0.0106	6.03×10^{-10}
rs364585	A/G	0.37	-0.0249,0.0038	4.28×10^{-10}
rs6016373	G/A	0.37	-0.0349,0.0037	7.95×10^{-19}
rs4253776	A/G	0.88	-0.0311,0.0059	3.35×10^{-8}
rs5763662	C/T	0.97	-0.0767,0.0121	1.19×10^{-8}
**HDL-C**				
rs4650994	A/G	0.48	-0.0210,0.0034	6.70×10^{-9}

rs12133576	G/A	0.65	-0.0243,0.0035	6.15×10^{-11}
rs1689797	A/C	0.30	-0.0358,0.0036	2.85×10^{-21}
rs4660293	G/A	0.24	-0.0353,0.004	2.86×10^{-18}
rs12748152	T/C	0.07	-0.0506,0.0062	9.74×10^{-16}
rs1047891	A/C	0.30	-0.0269,0.0039	8.73×10^{-10}
rs1515110	T/G	0.62	-0.0323,0.0035	8.04×10^{-18}
rs2290547	A/G	0.21	-0.0297,0.0046	3.69×10^{-9}
rs6805251	C/T	0.62	-0.0200,0.0035	1.33×10^{-8}
rs13076253	C/A	0.15	-0.0283,0.0048	4.96×10^{-9}
rs687339	T/C	0.77	-0.0316,0.0042	7.11×10^{-13}
rs2606736	T/C	0.61	-0.0246,0.0043	4.80×10^{-8}
rs10019888	G/A	0.16	-0.0270,0.0046	4.90×10^{-8}
rs3822072	A/G	0.49	-0.0251,0.0034	4.06×10^{-12}
rs2602836	G/A	0.57	-0.0192,0.0034	4.96×10^{-8}
rs13107325	T/C	0.08	-0.0708,0.0078	1.06×10^{-15}
rs6450176	A/G	0.28	-0.0254,0.0039	6.87×10^{-10}
rs998584	A/C	0.51	-0.0260,0.0038	2.27×10^{-11}
rs1936800	T/C	0.47	-0.0200,0.0034	3.05×10^{-10}
rs9457931	G/A	0.07	-0.0552,0.0073	7.30×10^{-13}
rs3861397	G/A	0.34	-0.0240,0.0036	8.40×10^{-11}
rs1980493	C/T	0.12	-0.0318,0.0048	3.76×10^{-10}
rs205262	G/A	0.27	-0.0283,0.0039	3.88×10^{-13}
rs4142995	T/G	0.38	-0.0263,0.0037	9.36×10^{-12}
rs17173637	C/T	0.10	-0.0363,0.0057	1.90×10^{-8}
rs10087900	A/G	0.44	-0.0231,0.0036	2.17×10^{-9}
rs1883025	T/C	0.24	-0.0698,0.0041	1.50×10^{-65}
rs12412743	T/C	0.15	-0.0291,0.0045	1.31×10^{-9}
rs2250802	A/G	0.68	-0.0340,0.0038	2.02×10^{-17}
rs102275	C/T	0.37	-0.0391,0.0035	6.40×10^{-28}

rs499974	A/C	0.18	-0.0263,0.0044	1.12×10^{-8}
rs11065987	G/A	0.42	-0.0222,0.0035	1.23×10^{-9}
rs838876	G/A	0.67	-0.0493,0.0039	7.32×10^{-33}
rs7306660	A/G	0.37	-0.0345,0.0036	3.34×10^{-19}
rs4983559	A/G	0.62	-0.0197,0.0036	9.57×10^{-9}
rs492571	C/T	0.04	-0.0663,0.0090	1.27×10^{-12}
rs2241770	C/T	0.10	-0.0989,0.0057	6.78×10^{-60}
rs4148005	G/T	0.30	-0.0283,0.0036	5.74×10^{-14}
rs6567160	C/T	0.23	-0.0257,0.0041	2.92×10^{-9}
rs737337	C/T	0.07	-0.0565,0.0061	4.56×10^{-17}
rs2075650	G/A	0.13	-0.0554,0.0051	9.72×10^{-26}
rs4465830	G/A	0.20	-0.0597,0.0044	5.17×10^{-40}
rs6031587	T/C	0.07	-0.0488,0.0074	1.92×10^{-9}
rs181360	G/T	0.20	-0.0376,0.0042	9.24×10^{-18}
rs12145743	T/G	0.67	-0.0203,0.0036	1.80×10^{-8}
rs4846914	G/A	0.42	-0.0479,0.0034	3.51×10^{-41}
rs12740374	G/T	0.79	-0.0343,0.0041	1.69×10^{-15}
rs2642438	A/G	0.25	-0.0303,0.0039	7.78×10^{-14}
rs676210	G/A	0.77	-0.0660,0.0040	2.34×10^{-54}
rs7607980	T/C	0.85	-0.0447,0.0052	1.81×10^{-15}
rs13099479	G/A	0.91	-0.0360,0.0062	1.82×10^{-8}
rs2013208	C/T	0.49	-0.0254,0.0036	8.92×10^{-12}
rs17145738	C/T	0.88	-0.0408,0.0053	4.95×10^{-13}
rs4917014	T/G	0.66	-0.0222,0.0036	1.03×10^{-8}
rs702485	A/G	0.55	-0.0243,0.0034	6.45×10^{-12}
rs11765979	A/C	0.54	-0.0412,0.0048	3.11×10^{-17}
rs1866956	C/T	0.32	-0.0217,0.0037	7.96×10^{-10}
rs4240624	G/A	0.08	-0.0818,0.0058	1.32×10^{-45}
rs2293889	T/G	0.41	-0.0312,0.0035	4.27×10^{-17}

rs10808546	C/T	0.55	-0.0409,0.0034	4.11×10^{-30}
rs13702	T/C	0.69	-0.1058,0.0038	1.30×10^{-160}
rs11789603	C/T	0.91	-0.0600,0.0060	3.69×10^{-21}
rs2066714	T/C	0.88	-0.0453,0.0071	7.26×10^{-10}
rs686030	C/A	0.14	-0.0550,0.0049	4.29×10^{-27}
rs10761771	T/C	0.53	-0.0198,0.0034	4.12×10^{-9}
rs970548	A/C	0.72	-0.0258,0.0039	1.71×10^{-10}
rs964184	G/C	NA	-0.1065,0.0071	6.09×10^{-48}
rs7112577	C/G	NA	-0.0826,0.0129	2.34×10^{-10}
rs3847502	C/A	0.69	-0.0480,0.0036	3.31×10^{-38}
rs12801636	G/A	0.78	-0.0235,0.0042	3.15×10^{-8}
rs2454722	A/G	0.85	-0.0351,0.0044	3.31×10^{-14}
rs4379922	T/C	0.65	-0.0247,0.0036	9.56×10^{-12}
rs3741414	C/T	0.81	-0.0296,0.0040	6.10×10^{-14}
rs11045163	A/G	0.59	-0.0217,0.0035	3.20×10^{-9}
rs2241210	A/G	0.45	-0.0332,0.0035	2.49×10^{-20}
rs10468017	C/T	0.72	-0.1179,0.0038	1.20×10^{-188}
rs424346	C/T	0.95	-0.0679,0.0113	4.84×10^{-8}
rs633695	A/G	0.75	-0.0885,0.0054	7.82×10^{-58}
rs9989419	A/G	0.41	-0.1473,0.0036	1.00×10^{-200}
rs16965220	C/A	0.70	-0.0219,0.0037	7.91×10^{-9}
rs16942887	G/A	0.87	-0.0831,0.0051	8.28×10^{-54}
rs2925979	T/C	0.30	-0.0351,0.0037	1.32×10^{-19}
rs1877031	G/A	0.32	-0.0336,0.0036	1.20×10^{-19}
rs4969178	A/G	0.37	-0.0263,0.0035	1.53×10^{-12}
rs4939883	T/C	0.18	-0.0799,0.0045	1.80×10^{-66}
rs2278236	G/A	0.46	-0.0331,0.0035	3.18×10^{-18}
rs2288912	C/G	0.50	-0.0297,0.0036	7.15×10^{-15}
rs103294	C/T	0.81	-0.0523,0.0044	3.99×10^{-30}

rs731839	G/A	0.34	-0.0220,0.0037	3.44×10^{-9}
**TG**				
rs4587594	A/G	0.31	-0.0694,0.0035	3.50×10^{-82}
rs1321257	A/G	0.59	-0.0402,0.0034	5.99×10^{-31}
rs1260326	C/T	0.59	-0.1148,0.0034	1.00×10^{-200}
rs13389219	T/C	0.41	-0.0271,0.0034	2.60×10^{-15}
rs676210	A/G	0.23	-0.0733,0.0039	3.28×10^{-71}
rs10440120	A/C	0.17	-0.0306,0.0044	5.34×10^{-11}
rs634869	C/T	0.56	-0.0272,0.0033	1.78×10^{-14}
rs2239520	A/G	0.37	-0.0236,0.0037	4.14×10^{-10}
rs287621	C/T	0.73	-0.0222,0.0037	7.67×10^{-9}
rs38855	G/A	0.47	-0.0187,0.0033	2.11×10^{-8}
rs11974409	G/A	0.19	-0.0899,0.0042	1.36×10^{-100}
rs12678919	G/A	0.12	-0.1702,0.0056	1.82×10^{-199}
rs4738684	G/A	0.64	-0.0205,0.0035	8.82×10^{-9}
rs2954022	A/C	0.47	-0.0780,0.0033	2.23×10^{-113}
rs1832007	G/A	0.13	-0.0327,0.0047	1.72×10^{-12}
rs2068888	A/G	0.49	-0.0241,0.0034	1.68×10^{-11}
rs10761762	C/T	0.47	-0.0270,0.0033	1.06×10^{-17}
rs10501321	C/T	0.31	-0.0216,0.0035	1.41×10^{-8}
rs948690	C/T	0.30	-0.0306,0.0052	6.57×10^{-9}
rs7350481	C/T	0.90	-0.2254,0.0066	1.00×10^{-200}
rs11613352	T/C	0.19	-0.0280,0.0039	9.40×10^{-14}
rs11057408	T/G	0.36	-0.0258,0.0035	2.05×10^{-12}
rs2043085	C/T	0.63	-0.0327,0.0034	7.81×10^{-20}
rs588136	T/C	0.79	-0.0495,0.0041	3.37×10^{-30}
rs749671	A/G	0.39	-0.0211,0.0034	6.11×10^{-10}
rs247616	T/C	0.29	-0.0393,0.0037	1.12×10^{-25}
rs3198697	T/C	0.38	-0.0198,0.0034	2.21×10^{-8}

rs7248104	A/G	0.42	-0.0222,0.0034	5.04×10^{-10}
rs10401969	C/T	0.07	-0.1210,0.0065	9.70×10^{-70}
rs731839	A/G	0.66	-0.0224,0.0036	2.65×10^{-9}
rs6029143	T/C	0.06	-0.0388,0.0071	4.93×10^{-8}
rs4810479	T/C	0.71	-0.0474,0.0038	2.07×10^{-34}
rs12748152	C/T	0.93	-0.0372,0.0059	1.10×10^{-9}
rs17513135	C/T	0.77	-0.0220,0.0039	1.63×10^{-8}
rs2972146	G/T	0.38	-0.0281,0.0034	2.97×10^{-15}
rs645040	G/T	0.23	-0.0293,0.0040	1.83×10^{-12}
rs6831256	A/G	0.59	-0.0258,0.0035	1.60×10^{-12}
rs442177	G/T	0.45	-0.0309,0.0033	1.32×10^{-18}
rs6882076	T/C	0.33	-0.0286,0.0035	1.51×10^{-15}
rs9686661	C/T	0.82	-0.0379,0.0044	2.54×10^{-16}
rs998584	C/A	0.49	-0.0293,0.0037	3.42×10^{-15}
rs719726	C/T	0.47	-0.0199,0.0035	2.49×10^{-8}
rs2665357	A/C	0.49	-0.0212,0.0033	8.33×10^{-10}
rs2247056	T/C	0.22	-0.0378,0.0039	3.86×10^{-21}
rs4719841	A/G	0.62	-0.0232,0.0034	8.86×10^{-11}
rs6995541	A/G	0.68	-0.0265,0.0037	1.34×10^{-12}
rs12676857	T/C	0.85	-0.0332,0.0046	7.29×10^{-12}
rs2250802	G/A	0.32	-0.0230,0.0037	1.21×10^{-10}
rs174535	T/C	0.64	-0.0470,0.0034	1.73×10^{-41}
rs12280753	C/T	0.93	-0.1931,0.0064	1.2×10^{-179}
rs16948098	G/A	0.96	-0.0800,0.0089	4.84×10^{-17}
rs8077889	A/C	0.76	-0.0252,0.0042	9.88×10^{-9}
rs3760627	T/C	0.53	-0.0189,0.0034	5.29×10^{-9}
rs439401	T/C	0.38	-0.0659,0.0038	1.42×10^{-66}
rs3761445	G/A	0.39	-0.0232,0.0034	8.06×10^{-12}
**TC**				

rs11591147	T/G	0.02	-0.3341,0.0173	8.83×10^{-86}
rs4988235	A/G	0.52	-0.0308,0.004	3.97×10^{-14}
rs17526895	G/A	0.08	-0.042,0.0067	5.78×10^{-9}
rs2287623	A/G	0.60	-0.0273,0.0036	4.09×10^{-12}
rs9306897	C/T	0.70	-0.0488,0.0037	7.51×10^{-37}
rs780093	C/T	0.59	-0.0515,0.0036	2.59×10^{-42}
rs6544713	C/T	0.71	-0.0773,0.004	1.69×10^{-81}
rs6709904	G/A	0.11	-0.0545,0.0083	8.39×10^{-10}
rs13315871	A/G	0.08	-0.0355,0.0061	3.48×10^{-8}
rs7616006	G/A	0.44	-0.0315,0.0036	8.41×10^{-17}
rs7640978	T/C	0.11	-0.0376,0.0066	1.66×10^{-8}
rs6818397	G/T	0.59	-0.0254,0.0039	9.51×10^{-11}
rs2814982	T/C	0.11	-0.0441,0.0057	3.68×10^{-15}
rs3757354	T/C	0.21	-0.0348,0.0042	2.22×10^{-15}
rs11153594	T/C	0.39	-0.029,0.0036	1.27×10^{-14}
rs1800562	A/G	0.05	-0.0565,0.0077	1.91×10^{-12}
rs9376090	C/T	0.27	-0.0254,0.004	2.60×10^{-09}
rs2737252	A/G	0.26	-0.0331,0.0039	1.63×10^{-16}
rs2954029	T/A	0.47	-0.0622,0.0035	2.42×10^{-65}
rs10088180	G/A	0.68	-0.0228,0.004	6.02×10^{-10}
rs4738684	G/A	0.65	-0.0392,0.0037	1.12×10^{-23}
rs1883025	T/C	0.24	-0.0671,0.0042	5.75×10^{-53}
rs3780181	G/A	0.05	-0.0442,0.0071	6.67×10^{-10}
rs12412743	T/C	0.15	-0.0298,0.0047	6.98×10^{-10}
rs2255141	G/A	0.68	-0.0314,0.0039	6.51×10^{-16}
rs1535	G/A	0.36	-0.0497,0.0037	8.62×10^{-39}
rs964184	C/G	NA	-0.1214,0.0076	2.84×10^{-55}
rs4883201	G/A	0.11	-0.035,0.0056	1.74×10^{-9}
rs6573778	C/T	0.53	-0.0263,0.0039	2.96×10^{-11}

rs2886232	C/T	0.88	-0.0358,0.0062	$3.87×10^{-8}$
rs314253	C/T	0.34	-0.0233,0.0037	$2.81×10^{-10}$
rs2738459	C/A	0.44	-0.0387,0.0057	$2.11×10^{-11}$
rs2228603	T/C	0.07	-0.1217,0.0069	$1.05×10^{-62}$
rs281393	T/C	0.37	-0.0322,0.0055	$4.26×10^{-8}$
rs6511720	T/G	0.10	-0.1851,0.0059	$1.00×10^{-200}$
rs7412	T/C	0.07	-0.3736,0.0096	$1.00×10^{-200}$
rs6016373	G/A	0.37	-0.0319,0.0036	$1.00×10^{-17}$
rs1800961	T/C	0.03	-0.1062,0.0101	$1.34×10^{-24}$
rs2277862	T/C	0.13	-0.0349,0.0052	$5.26×10^{-11}$
rs181360	G/T	0.20	-0.0278,0.0043	$7.32×10^{-10}$
rs138777	G/A	0.65	-0.0214,0.0037	$4.74×10^{-8}$
rs11802413	C/T	0.46	-0.0287,0.0035	$1.58×10^{-14}$
rs646776	C/T	0.21	-0.1272,0.0042	$4.78×10^{-187}$
rs558971	A/G	0.47	-0.0398,0.0036	$7.02×10^{-28}$
rs2642438	A/G	0.25	-0.037,0.004	$1.28×10^{-18}$
rs7534572	C/G	0.31	-0.0629,0.0055	$3.60×10^{-28}$
rs6603981	C/T	0.19	-0.0351,0.0043	$7.85×10^{-15}$
rs7551981	G/T	0.41	-0.0358,0.0037	$7.50×10^{-22}$
rs11694172	A/G	0.78	-0.0277,0.0041	$1.95×10^{-9}$
rs2030746	C/T	0.60	-0.0199,0.0037	$3.60×10^{-8}$
rs11563251	C/T	0.87	-0.0368,0.0059	$1.27×10^{-9}$
rs515135	T/C	0.22	-0.1238,0.0046	$6.38×10^{-151}$
rs4530754	G/A	0.42	-0.0228,0.0035	$1.68×10^{-9}$
rs12916	T/C	0.57	-0.0684,0.0036	$4.55×10^{-74}$
rs6882076	T/C	0.33	-0.0508,0.0037	$5.35×10^{-41}$
rs9272775	T/C	0.72	-0.0317,0.0055	$2.13×10^{-8}$
rs2315065	C/A	0.91	-0.1102,0.0158	$1.10×10^{-11}$
rs9391858	A/G	0.81	-0.0495,0.005	$7.20×10^{-22}$

rs112201728	C/T	0.94	-0.0581,0.0099	1.20×10^{-8}
rs11753995	G/A	0.85	-0.0489,0.0048	1.84×10^{-23}
rs1997243	A/G	0.87	-0.0332,0.005	2.72×10^{-10}
rs12670798	T/C	0.78	-0.0364,0.0041	9.48×10^{-17}
rs2073547	A/G	0.81	-0.0456,0.0047	3.83×10^{-21}
rs9987289	A/G	0.08	-0.0842,0.0063	1.84×10^{-36}
rs7832643	G/T	0.60	-0.0289,0.0037	3.12×10^{-13}
rs581080	G/C	0.18	-0.0377,0.0047	1.02×10^{-13}
rs11789603	C/T	0.91	-0.0427,0.0062	1.44×10^{-11}
rs2066714	T/C	0.88	-0.0442,0.0076	1.14×10^{-8}
rs579459	T/C	0.79	-0.062,0.0044	8.83×10^{-42}
rs10904908	A/G	0.55	-0.025,0.0036	2.60×10^{-11}
rs10900221	G/A	0.73	-0.0255,0.0041	7.96×10^{-9}
rs4752805	A/G	0.75	-0.0251,0.0041	1.62×10^{-9}
rs10832962	C/T	0.28	-0.0315,0.0039	1.54×10^{-14}
rs11220462	G/A	0.86	-0.0474,0.0058	5.49×10^{-15}
rs3184504	T/C	0.47	-0.0318,0.0037	1.62×10^{-17}
rs10773003	G/A	0.91	-0.0369,0.0058	4.08×10^{-9}
rs2244608	A/G	0.66	-0.0313,0.0037	9.62×10^{-18}
rs10468017	C/T	0.72	-0.0617,0.004	7.23×10^{-48}
rs633695	A/G	0.72	-0.0433,0.0058	1.05×10^{-14}
rs2000999	G/A	0.82	-0.0617,0.0044	6.80×10^{-41}
rs247616	C/T	0.71	-0.0499,0.004	4.47×10^{-32}
rs6504872	C/T	0.53	-0.025,0.0035	6.99×10^{-12}
rs2156552	A/T	0.18	-0.057,0.0047	1.25×10^{-31}
rs8103315	C/A	0.86	-0.0422,0.0055	5.94×10^{-15}
rs75687619	G/T	0.98	-0.1592,0.0153	3.61×10^{-22}
rs386003	G/T	0.80	-0.0344,0.0058	4.52×10^{-8}
rs2235367	A/G	0.54	-0.0357,0.0035	7.22×10^{-25}

rs4253772	C/T	0.88	-0.0322,0.0058	9.85×10^{-9}
**ApoA**				
rs4860951	T/A	0.35	0.0737,0.0132	3.29×10^{-8}
rs144064722	G/A	0.03	0.2037,0.0350	8.30×10^{-9}
rs75835816	G/C	0.98	0.2210,0.0388	1.67×10^{-8}
rs1461729	G/A	0.86	0.0864,0.0152	1.77×10^{-8}
rs1883025	C/T	0.81	0.0798,0.0133	2.86×10^{-9}
rs174594	A/C	0.59	0.0717,0.0105	1.32×10^{-11}
rs261291	C/T	0.37	0.1443,0.0109	2.58×10^{-39}
rs11632618	A/G	0.06	0.1741,0.0241	7.76×10^{-13}
rs73424577	G/A	0.04	0.1860,0.0302	9.78×10^{-10}
rs247617	A/C	0.29	0.1972,0.0117	7.90×10^{-63}
rs6507939	C/A	0.84	0.1084,0.0143	5.03×10^{-14}
**ApoB**				
rs629301	T/G	0.78	0.0901,0.0122	3.56×10^{-13}
rs1367117	A/G	0.29	0.1089,0.0112	9.99×10^{-22}
rs182695896	C/A	0.02	0.2365,0.0418	2.49×10^{-8}
rs144064722	G/A	0.03	0.1990,0.0351	2.29×10^{-8}
rs10056811	A/G	0.34	0.0858,0.0106	1.35×10^{-15}
rs635634	T/C	0.20	0.0740,0.0126	7.71×10^{-9}
rs1081105	C/A	0.02	0.2229,0.0393	2.32×10^{-8}
rs1883711	C/G	0.06	0.1441,0.0253	1.95×10^{-8}
rs190934192	G/A	0.98	0.3197,0.0408	1.30×10^{-14}
rs11591147	G/T	0.97	0.4379,0.0353	2.50×10^{-34}
rs3005923	G/A	0.97	0.2829,0.0369	4.73×10^{-14}
rs2495477	A/G	0.58	0.0618,0.0110	3.14×10^{-8}
rs1260326	T/C	0.36	0.0667,0.0103	2.51×10^{-10}
rs6756629	G/A	0.92	0.1132,0.0185	1.90×10^{-9}
rs4722043	G/C	0.58	0.0659,0.0106	1.17×10^{-9}

rs115849089	G/A	0.89	0.0995,0.0171	1.04×10^{-8}
rs2980875	A/G	0.52	0.0696,0.0099	6.68×10^{-12}
rs964184	G/C	0.14	0.1657,0.0142	2.58×10^{-30}
rs7412	C/T	0.94	0.4275,0.0259	4.39×10^{-59}
rs142130958	G/A	0.89	0.1996,0.0167	7.78×10^{-32}
rs150617279	T/A	0.89	0.1123,0.0176	3.97×10^{-10}

Abbreviations: LDL-C: low-density lipoprotein cholesterol; HDL-C: high-density lipoprotein cholesterol; TC: total cholesterol; TG: triglyceride; ApoA: Apolipoprotein A; ApoB: Apolipoprotein B; EA: effect allele; NEA: non-effect allele; EAF: effect allele frequency.

Supplementary Table 3 *F*-statistics estimates for genetic instruments, variance explained by genetic instruments and statistical power (%) estimates for primary analyses.

Outcome	Males	Females	Overall
HMGCR (F=128.5, r^2=0.0040)	0.45	0.30	0.22
NPC1L1 (F=71.1, r^2=0.0013)	0.18	0.13	0.10
PCSK9 (F=141.9, r^2=0.0079)	0.73	0.52	0.38
CETP (F=64.2, r^2=0.0035)	0.40	0.27	0.19
LDLR (F=181.2, r^2=0.0126)	0.90	0.72	0.55
APOB (F=369.6, r^2=0.0151)	0.94	0.80	0.62
LDL-C (F=131.1, r^2=0.0740)	1	1	1
HDL-C (F=103.3, r^2=0.0535)	1	1	0.99
TG (F=153.6, r^2=0.0481)	1	1	0.98
TC (F=123.8, r^2=0.0710)	1	1	1
ApoA (F=73.5, r^2=0.0435)	1	1	0.97
ApoB (F=68.1, r^2=0.0752)	1	1	1

* Power calculations represent statistical power to detect an odds ratio of OR 0.50 per 1 SD (38.67 mg/dL) reduction in LDL-cholesterol at type I error of 5%.

Supplementary Table 4 Association of Genetically Proxied Inhibition of *HMGCR*, *NPC1L1*, *PCSK9*, *CETP*, *LDLR*, *APOB* with Overall and Sex-specific Renal Cell Carcinoma Risk after adjusted for weakly linkage disequilibrium (r^2 ≤0.20) among variants.

Targets	Study	N_{SNPs}	IVW				Weighted median		MR-Egger	
			OR (95% CI)	*P*	*q*-value	$P_{difference}$	OR (95% CI)	*P*	OR (95% CI)	*P*
HMGCR	Male	5	1.251 [0.562-2.786]	0.583	0.600	0.70	1.377 [0.637-2.979]	0.416	3.115 [0.050-195.6]	0.591
	Female	5	0.972 [0.355-2.659]	0.956	0.711		0.945 [0.352-2.539]	0.910	0.231 [0.002-27.32]	0.548
	Overall	5	1.418 [0.288-6.994]	0.668	0.632		1.426 [0.291-6.985]	0.662	0.104 [0.000-214.9]	0.561
	CAD	5	0.598 [0.426-0.840]	0.003	-		0.573 [0.413-0.794]	8.44×10^{-4}	0.263 [0.065-1.057]	0.060
NPC1L1	Male	4	0.821 [0.205-3.288]	0.781	0.667	0.83	0.819 [0.223-3.007]	0.764	0.239 [0.000-204.3]	0.678
	Female	4	0.642 [0.115-3.577]	0.613	0.612		1.110 [0.191-6.451]	0.907	0.585 [0.000-1224]	0.891
	Overall	3	0.215 [0.016-2.860]	0.245	0.386		0.268 [0.022-3.217]	0.299	0.381 [0.000-5.94×10^{5}]	0.895
	CAD	4	0.447 [0.202-0.987]	0.046	-		0.466 [0.248-0.875]	0.018	3.074 [0.082-115.6]	0.544
PCSK9	Male	8	2.198 [1.241-3.893]	0.007	**0.045**	0.10	2.262 [1.154-4.433]	0.017	1.334 [0.144-12.34]	0.800
	Female	8	0.956 [0.424-2.157]	0.914	0.701		0.779 [0.318-1.908]	0.584	0.843 [0.041-17.17]	0.911
	Overall	8	1.547 [0.535-4.476]	0.421	0.520		1.614 [0.451-5.779]	0.462	0.604 [0.009-39.59]	0.813
	CAD	7	0.559 [0.432-0.724]	1.04×10^{-5}	-		0.557 [0.401-0.773]	4.59×10^{-4}	0.430 [0.128-1.447]	0.173
LDLR	Male	11	1.356 [0.850-2.161]	0.201	0.341	0.23	1.410 [0.910-2.184]	0.124	1.665 [0.852-3.256]	0.136

	Female	11	0.854 [0.474-1.538]	0.598	0.606		0.803 [0.450-1.432]	0.458	0.754 [0.319-1.787]	0.522
	Overall	11	1.092 [0.485-2.454]	0.832	0.681		0.967 [0.414-2.258]	0.938	0.783 [0.165-3.719]	0.759
	CAD	10	0.478 [0.378-0.605]	7.82×10^{-10}	-		0.491 [0.381-0.632]	3.25×10^{-8}	0.595 [0.415-0.855]	0.005
APOB	Male	7	0.590 [0.324-1.073]	0.084	0.240	0.04	0.571 [0.357-0.915]	0.020	0.209 [0.063-0.702]	0.011
	Female	7	1.423 [0.838-2.617]	0.192	0.336		1.375 [0.798-2.369]	0.251	1.735 [0.378-7.961]	0.479
	Overall	9	0.509 [0.208-1.247]	0.140	0.301		0.468 [0.197-1.109]	0.085	0.466 [0.064-3.390]	0.451
	CAD	14	0.720 [0.592-0.876]	1.03×10^{-3}	-		0.706 [0.586-0.849]	2.26×10^{-4}	0.565 [0.379-0.844]	0.005
CETP	Male	8	2.164 [0.766-6.112]	0.145	0.305	0.41	2.535 [1.013-6.341]	0.047	0.543 [0.036-8.112]	0.658
	Female	8	4.313 [1.194-15.58]	0.026	0.112		3.801 [1.175-12.30]	0.026	3.042 [0.071-129.9]	0.561
	Overall	8	18.76 [2.446-143.9]	4.79×10^{-4}	**0.006**		11.38 [1.857-69.77]	8.57×10^{-3}	1.498 [0.001-2148]	0.913
	CAD	6	0.401 [0.212-0.759]	0.005	**-**		0.435 [0.239-0.792]	6.49×10^{-3}	0.246 [0.038-1.576]	0.139

Abbreviation: IVW, inverse-variance weighted; OR, odds ratio; CAD: cardiovascular disease; N_{out}: Number of participants in the renal cell carcinoma genome-wide association study. N_{SNPs}: Numbers of SNPs in each instrument variable. *q*-values were used to indicate the FDR-corrected *P* values. $P_{difference}$ for difference between sex-specific effect estimates.

Supplementary Table 5 Mendelian randomization results of circulating lipid traits with overall and sex-specific renal cell carcinoma risk.

Trait	Method	Overall			Male			Female		
		N_{SNPs}	OR (95% CI)	*P*	N_{SNPs}	OR (95% CI)	*P*	N_{SNPs}	OR (95% CI)	*P*
LDL-C	IVW	67	0.83 [0.57-1.22]	0.350	67	0.87 [0.71-1.06]	0.16	66	1.11 [0.85-1.46]	0.43
	Weighted median	67	1.01 [0.56-1.82]	0.974	67	0.88 [0.65-1.19]	0.42	66	1.19 [0.81-1.72]	0.37
	Weighted mode	67	1.09 [0.61-1.96]	0.767	67	0.82 [0.56-1.19]	0.24	66	1.09 [0.75-1.56]	0.66
	MR Egger	67	1.12 [0.55-2.28]	0.761	67	0.81 [0.57-1.16]	0.26	66	1.03 [0.62-1.71]	0.90
	MR PRESSO	67	NA	NA	67	NA	NA	66	NA	NA
HDL-C	IVW	82	1.15 [0.78-1.69]	0.473	84	1.22 [0.97-1.53]	0.082	80	1.05 [0.81-1.37]	0.72
	Weighted median	82	1.73 [0.91-3.29]	0.093	84	1.17 [0.85-1.61]	0.35	80	1.19 [0.83-1.72]	0.34
	Weighted mode	82	1.76 [0.88-3.54]	0.114	84	1.14 [0.82-1.58]	0.43	80	1.18 [0.81-1.72]	0.40
	MR Egger	82	1.60 [0.78-3.26]	0.202	84	1.35 [0.88-2.07]	0.17	80	1.45 [0.89-2.37]	0.14
	MR PRESSO	82	NA	NA	84	1.16 [0.95-1.43]	0.15	80	NA	NA
TG	IVW	54	0.78 [0.50-1.22]	0.275	55	1.00 [0.81-1.23]	1.00	52	0.96 [0.74-1.26]	0.78
	Weighted median	54	1.01 [0.54-1.89]	0.581	55	0.93 [0.68-1.27]	0.64	52	1.07 [0.72-1.59]	0.73
	Weighted mode	54	0.99 [0.57-1.70]	0.963	55	0.93 [0.68-1.28]	0.67	52	1.02 [0.70-1.49]	0.91
	MR Egger	54	0.82 [0.40-1.67]	0.581	55	0.94 [0.67-1.32]	0.73	52	0.75 [0.49-1.14]	0.18

	MR PRESSO	54	NA	NA	55	NA	NA	52	NA	NA
TC	IVW	81	0.91 [0.66-1.27]	0.589	82	1.20 [1.00-1.44]	0.044	81	0.95 [0.75-1.20]	0.68
	Weighted median	81	1.00 [0.60-1.68]	0.988	82	1.05 [0.79-1.38]	0.746	81	0.90 [0.65-1.25]	0.52
	Weighted mode	81	0.95 [0.56-1.62]	0.852	82	1.00 [0.70-1.42]	0.994	81	0.87 [0.61-1.22]	0.42
	MR Egger	81	1.06 [0.61-1.82]	0.843	82	1.26 [0.93-1.72]	0.135	81	0.88 [0.59-1.31]	0.53
	MR PRESSO	81	NA	NA	82	NA	NA	81	NA	NA
ApoA	IVW	8	1.20 [0.70-2.07]	0.502	8	1.18 [0.88-1.58]	0.28	7	1.25 [0.92-1.69]	0.16
	Weighted median	8	1.64 [0.88-3.06]	0.117	8	1.19 [0.88-1.60]	0.25	7	1.18 [0.83-1.69]	0.36
	Weighted mode	8	1.48 [0.81-2.72]	0.247	8	1.20 [0.88-1.64]	0.29	7	1.18 [0.78-1.80]	0.47
	MR Egger	8	1.14 [0.24-5.50]	0.873	8	1.49 [0.64-3.46]	0.39	7	0.89 [0.38-2.09]	0.80
	MR PRESSO	8	NA	NA	8	NA	NA	7	NA	NA
ApoB	IVW	13	0.82 [0.56-1.19]	0.294	12	1.24 [0.95-1.62]	0.12	12	0.89 [0.64-1.23]	0.47
	Weighted median	13	0.79 [0.48-1.32]	0.374	12	1.43 [1.07-1.91]	0.014	12	0.76 [0.53-1.09]	0.14
	Weighted mode	13	0.78 [0.47-1.29]	0.343	12	1.46 [1.10-1.94]	0.025	12	0.78 [0.55-1.10]	0.18
	MR Egger	13	0.80 [0.41-1.57]	0.531	12	1.89 [1.28-2.80]	0.009	12	0.71 [0.40-1.26]	0.27
	MR PRESSO	13	NA	NA	12	NA	NA	12	NA	NA

Supplementary Table 6 I^2_{GX} statistics to assess violation of the "NO Measurement Error" (NOME) assumption for instruments used in MR-Egger regression.

	Male		Female	
Exposure	Isq unweighted	Isq weighted	Isq unweighted	Isq weighted
Drug target				
HMGCR	0.89	0.96	0.89	0.96
NPC1L1	0.71	0.72	0.71	0.73
PCSK9	0.89	0.83	0.89	0.83
CETP	0.76	0.78	0.76	0.77
LDLR	0.99	0.99	0.99	0.99
APOB	0.98	0.98	0.98	0.98
Lipid traits				
LDL-C	0.97	0.97	0.97	0.97
HDL-C	0.96	0.96	0.97	0.96
TG	0.99	0.99	0.99	0.99
TC	0.98	0.97	0.98	0.97
ApoA	0.98	0.98	0.98	0.98
ApoB	0.99	0.99	0.99	0.99

Supplementary Table 7 SIMEX corrected MR-Egger estimates.

Exposure	Male		Female	
	Unweighted	Weighted	Unweighted	Weighted
Drug target				
HMGCR	7.39 [0.96-57.0]	3.93 [0.69-22.3]	0.07 [0.01-0.88]	0.16 [0.02-1.63]
NPC1L1	0.26 [0.00-16.3]	0.36 [0.00-1927]	7.91 [0.00-15176]	5.62 [0.00-19646]
PCSK9	1.43 [0.15-14.0]	1.34 [0.11-16.2]	0.75 [0.01-43.4]	0.66 [0.01-31.6]
CETP	0.23 [0.01-4.50]	0.54 [0.03-8.77]	16.1 [0.19-1346]	3.54 [0.07-173]
LDLR	1.70 [1.02-2.85]	1.76 [0.90-3.43]	0.78 [0.35-1.78]	0.81 [0.31-2.14]
APOB	0.15 [0.03-0.82]	0.16 [0.04-0.66]	1.74 [0.34-8.87]	1.66 [0.51-5.41]
Lipid traits				
LDL-C	1.30 [0.97-1.74]	1.23 [0.90-1.70]	1.01 [0.60-1.70]	1.00 [0.59-1.68]
HDL-C	0.51 [0.23-1.12]	0.45 [0.22-0.91]	0.34 [0.14-0.81]	0.39 [0.17-0.90]
TG	1.34 [0.90-2.00]	1.06 [0.76-1.50]	1.34 [0.90-2.00]	1.34 [0.90-2.00]
TC	0.74 [0.59-0.94]	0.81 [0.60-1.09]	1.15 [0.78-1.71]	1.15 [0.77-1.71]
ApoA	0.50 [0.18-1.45]	0.64 [0.26-1.57]	1.07 [0.43-2.61]	1.14 [0.56-2.32]
ApoB	0.55 [0.40-0.77]	0.51 [0.34-0.77]	1.53 [0.90-2.60]	1.43 [0.82-2.51]

Supplementary Table 8 Colocalization analysis of circulating LDL-C levels and risk of overall and sex-specific renal cell carcinoma for *PCSK9* and *CETP* variants.

Drug target	Gender	Causal variant	CLPP	Credible set posterior probability	LDL-C *P*-value	RCC *P*-value
PCSK9	Male	rs11206510	0.004	> 0.99	2.38×10^{-53}	0.046
CETP	Female	rs247616	0.004	> 0.99	2.57×10^{-37}	0.437

Supplementary Table 9 Univariable Mendelian randomization analyses assessed the association between genetically-proxied inhibition of *HMGCR*, *NPC1L1*, *PCSK9*, *CETP*, *LDLR*, *APOB* and previously reported risk factors for renal cell carcinoma.

		HMGCR		***NPC1L1***		***PCSK9***		***CETP***		***LDLR***		***APOB***	
**Proposed risk factor**	**N**	***β*/OR (95% CI)**	***P***	***β*/OR (95% CI)**	***P***	***β*/OR (95% CI)**	***P***	***β*/OR (95% CI)**	***P***	***β*/OR (95% CI)**	***P***	***β*/OR (95% CI)**	***P***
**Smoking**													
Smoking initiation age	341,427	-0.06 [-0.13, 0.01]	0.07	-0.07 [-0.18, 0.03]	0.16	-0.01 [-0.04, 0.02]	0.496	-0.06 [-0.16, 0.04]	0.232	0.01 [-0.02, 0.05]	0.450	-0.01 [-0.04, 0.03]	0.716
Cigarettes per day	337,334	0.03 [-0.12, 0.17]	0.72	0.33 [0.12, 0.53]	0.002	0.05 [-0.01, 0.11]	0.123	0.03 [-0.13, 0.19]	0.730	-0.08 [-0.15, -0.02]	0.013	0.07 [-0.01, 0.14]	0.068
Smoking initiation	1,232,091	1.09 [0.96-1.23]	0.18	0.94 [0.82-1.07]	0.35	1.01 [0.97-1.05]	0.585	0.97 [0.88-1.08]	0.593	1.00 [0.96-1.05]	0.943	1.03 [0.99-1.07]	0.132
Lifetime smoking index	462,690	0.03 [0.00-0.07]	0.055	0.08 [0.02, 0.13]	0.004	0.02 [0.00, 0.03]	0.028	-0.03 [-0.07, 0.01]	0.188	0.00 [-0.01, 0.02]	0.614	0.01 [-0.01, 0.03]	0.176
**Alcohol drinking**													
Drinks per week	335,394	0.00 [-0.06, 0.06]	0.99	-0.01 [-0.09, 0.06]	0.73	-0.01 [-0.03, 0.01]	0.543	0.04 [-0.02, 0.09]	0.222	-0.03 [-0.05, 0.00]	0.050	-0.01 [-0.03, 0.02]	0.550
**Anthropometric traits**													
Body mass index	681,275	0.26 [0.17-0.36]	4.32×10^{-8}	0.04 [-0.04, 0.11]	0.377	0.01 [-0.01, 0.04]	0.361	-0.06 [-0.11, -0.01]	0.017	0.02 [-0.02, 0.07]	0.311	0.04 [0.02-0.06]	1.13×10^{-4}
Waist-to-Hip Ratio	697,734	-0.01 [-0.09-0.07]	0.82	0.00 [-0.11, 0.11]	0.99	0.05 [0.03, 0.07]	7.26×10^{-6}	-0.05 [-0.10, 0.01]	0.085	0.01 [-0.02, 0.04]	0.401	0.01 [-0.01, 0.03]	0.521
Height	693,529	0.26 [0.17-0.36]	4.32×10^{-8}	0.04 [-0.04, 0.11]	0.377	0.01 [-0.02, 0.04]	0.424	-0.06 [-0.11, -0.01]	0.025	0.02 [-0.03, 0.06]	0.418	0.04 [0.02, 0.06]	1.75×10^{-5}
**Hypertension**													
Systolic pressure	757,601	-0.78 [-1.62, 0.05]	0.067	-0.51 [-2.64, 1.61]	0.64	-0.08 [-0.56, 0.41]	0.761	-1.31 [-2.19, -0.42]	0.004	-0.81 [-1.31, -0.31]	0.001	-0.24 [-0.76, 0.28]	0.366

Diastolic pressure	757,601	-1.06 [-1.83, -0.29]	0.007	-0.34 [-1.08, 0.41]	0.38	0.11 [-0.13, 0.36]	0.372	-0.59 [-1.10, -0.07]	0.025	0.22 [-0.22, 0.65]	0.326	0.15 [-0.06, 0.36]	0.158
**Diabetes**													
Fasting glucose	46,186	0.07 [-0.03-0.18]	0.17	0.13 [-0.03, 0.28]	0.105	-0.01 [-0.07, 0.06]	0.870	-0.07 [-0.24, 0.10]	0.426	0.00 [-0.05, 0.05]	0.957	0.05 [0.00, 0.10]	0.053
Fasting insulin	38,238	0.08 [-0.05-0.21]	0.23	-0.14 [-0.31, 0.02]	0.080	-0.03 [-0.11, 0.05]	0.487	-0.05 [-0.18, 0.08]	0.476	-0.02 [-0.07, 0.04]	0.576	0.02 [-0.03, 0.07]	0.576
Type 2 diabetes	149,821	1.13 [0.77-1.67]	0.532	2.69 [1.67-4.32]	4.29×10^{-5}	1.28 [1.09-1.51]	0.002	0.82 [0.54-1.24]	0.350	1.12 [0.97-1.30]	0.126	1.13 [0.98-1.29]	0.081
**Chronic kidney disease**	117,165	0.89 [0.62-1.27]	0.51	0.54 [0.29-1.01]	0.055	1.00 [0.69-1.44]	0.993	0.67 [0.41-1.09]	0.108	1.23 [1.03-1.47]	0.024	0.89 [0.70-1.14]	0.374

Supplementary Table 10 Association between genetically-proxied inhibition of *PCSK9*, *CETP* and renal cell carcinoma in males and females , adjusted for significant RCC risk factors identified by univariable MR analyses.

Exposure	N SNPs	Q-stat for instrument strength	*F*-stat	*Q*-stat for instrument validity	*P*-value for instrument validity	Method	OR	95% CI	*P*
MR analysis for WHR and genetically proxied PCSK9 inhibition with RCC risk in males									
WHR	310	10464.3	32.91	414.7	1.35×10^{-4}	IVW	1.20	0.86-1.67	0.28
						MR-Egger	1.25	0.90-1.75	0.19
LDL-C	8	1865.7	5.87			IVW	1.59	1.02-2.49	0.041
						MR-Egger	1.31	0.76-2.26	0.32
MR analysis for lifetime smoking index and genetically proxied PCSK9 inhibition with RCC risk in males									
LSI	59	2751.9	41.07	76.5	0.135	IVW	0.41	0.15-1.15	0.090
						MR-Egger	0.25	0.00-13.3	0.495
LDL-C	8	1290.9	19.27			IVW	1.73	1.02-2.93	0.042
						MR-Egger	1.61	0.76-3.42	0.211
MR analysis for type 2 diabetes and genetically proxied PCSK9 inhibition with RCC risk in males									
T2D	39	2609.4	34.07	39.4	0.667	IVW	1.03	0.92-1.15	0.659

						MR-Egger	NA	NA	NA
LDL-C	8	1601.2	55.52			IVW	2.07	1.33-3.24	0.001
						MR-Egger	1.82	1.08-3.08	0.025
MR analysis for BMI and genetically proxied CETP inhibition with RCC risk in females									
BMI	463	33983.7	72.15	511.6	0.080	IVW	0.76	0.57-1.02	0.064
						MR-Egger	0.56	0.26-1.22	0.145
LDL-C	8	2137.7	4.54			IVW	1.21	0.77-1.89	0.409
						MR-Egger	1.18	0.75-1.85	0.481
MR analysis for height and genetically proxied CETP inhibition with RCC risk in females									
Height	480	34745.4	71.20	526.9	0.092	IVW	1.43	1.08-1.91	0.013
						MR-Egger	1.88	0.88-4.04	0.106
LDL-C	8	2192.6	4.49			IVW	0.90	0.58-1.40	0.647
						MR-Egger	0.93	0.59-1.45	0.737
MR analysis for systolic pressure and genetically proxied CETP inhibition with RCC risk in females									
SBP	310	19525.1	69.74	340.7	0.153	IVW	2.27	1.22-4.22	0.010
						MR-Egger	2.39	1.26-4.52	0.008
LDL-C	8	1042.1	3.28			IVW	1.00	0.98-1.02	0.985

						MR-Egger	1.02	0.97-1.07	0.523
MR analysis for diastolic pressure and genetically proxied CETP inhibition with RCC risk in males									
DBP	321	26479.4	80.48	400.4	0.003	IVW	1.82	1.06-3.13	0.030
						MR-Egger	1.76	1.01-3.05	0.046
LDL-C	8	1527.9	4.64			IVW	1.01	0.97-1.04	0.765
						MR-Egger	0.98	0.90-1.07	0.666

Supplementary Table 11 Assessing heterogeneity of single nucleotide polymorphism (SNP) effect estimates in inverse-variance weighted (IVW) and MR-Egger regression.

Exposure	Male				Female			
	IVW *Q*	*P*	MR-Egger *Q*	*P*	IVW *Q*	*P*	MR-Egger *Q*	*P*
Drug-target								
HMGCR	1.73	0.79	1.23	0.75	2.26	0.69	1.49	0.68
NPC1L1	1.61	0.66	1.42	0.49	2.91	0.41	2.91	0.23
PCSK9	6.56	0.48	6.34	0.39	9.29	0.23	9.28	0.16
CETP	6.68	0.46	5.33	0.50	4.33	0.74	4.27	0.64
LDLR	12.51	0.25	11.77	0.23	12.35	0.26	12.19	0.20
APOB	11.92	0.06	7.77	0.17	4.00	0.68	3.90	0.56
Lipid traits								
LDL-C	50.71	0.92	50.51	0.91	78.05	0.13	77.91	0.11
HDL-C	115.95	0.01	115.52	0.01	94.50	0.11	91.80	0.14
TG	54.19	0.47	53.98	0.44	46.94	0.64	44.57	0.69
TC	89.61	0.24	89.43	0.22	97.04	0.09	96.75	0.09
ApoA	10.09	0.18	9.55	0.14	3.87	0.69	3.18	0.67
ApoB	17.61	0.09	10.56	0.39	16.69	0.12	15.38	0.12

Supplementary Table 12 Assessing directional pleiotropy through MR-Egger intercept.

Exposure	Male			Female		
	N_{SNPs}	OR (95% CI)	*P*	N_{SNPs}	OR (95% CI)	*P*
Drug-target						
HMGCR	5	1.07 [0.91-1.26]	0.47	5	0.90 [0.73-1.10]	0.38
NPC1L1	4	0.97 [0.77-1.21]	0.80	4	1.07 [0.81-1.42]	0.67
PCSK9	8	0.97 [0.85-1.11]	0.65	8	0.99 [0.83-1.19]	0.93
CETP	8	0.94 [0.84-1.05]	0.27	8	0.98 [0.84-1.15]	0.84
LDLR	11	1.02 [0.97-1.07]	0.42	11	0.99 [0.93-1.05]	0.71
APOB	7	0.90 [0.80-1.01]	0.07	7	1.02 [0.88-1.18]	0.78
Lipid traits						
LDL-C	69	1.00 [0.99-1.02]	0.66	68	1.00 [0.98-1.03]	0.74
HDL-C	86	0.99 [0.98-1.01]	0.58	82	0.98 [0.96-1.01]	0.13
TG	55	1.00 [0.99-1.02]	0.65	52	1.02 [0.99-1.04]	0.13
TC	84	1.00 [0.98-1.01]	0.69	83	1.00 [0.98-1.03]	0.63
ApoA	8	0.97 [0.87-1.08]	0.58	7	1.05 [0.94-1.17]	0.44
ApoB	12	0.94 [0.90-0.99]	0.03	13	1.03 [0.96-1.11]	0.38

(a) HMGCR

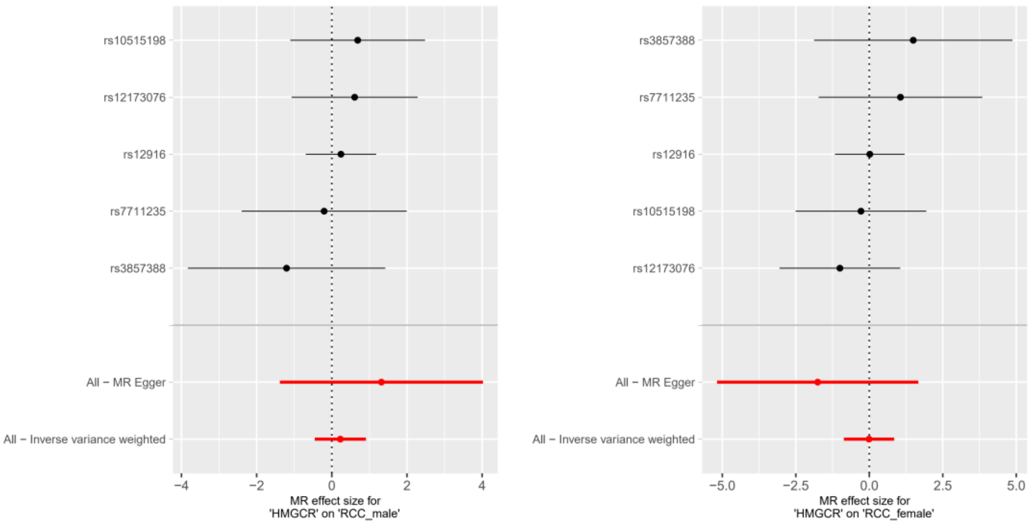

(b) NPC1L1

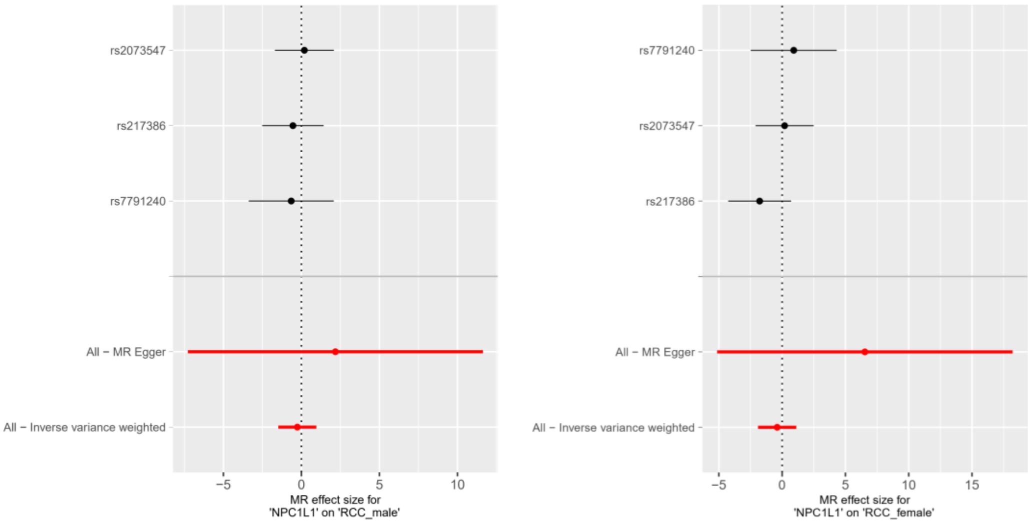

(c) LDLR

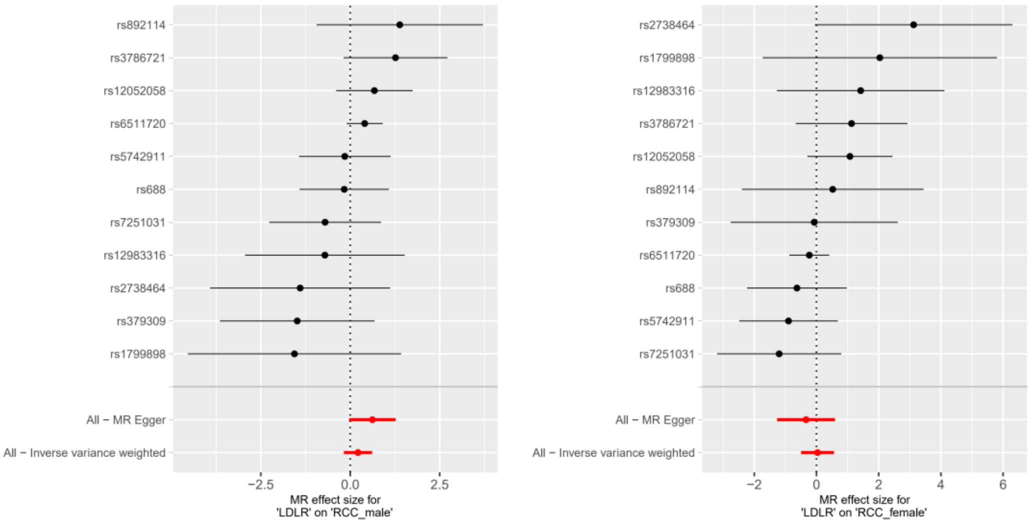

(d) APOB

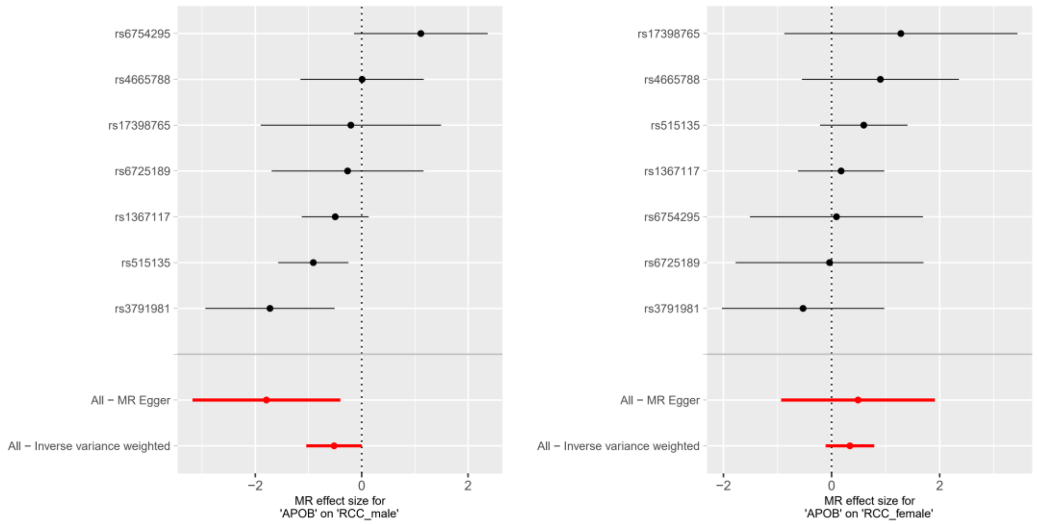

Supplementary Figure 2 Drug-target Mendelian randomization analysis single SNP plots.

(a) HMGCR

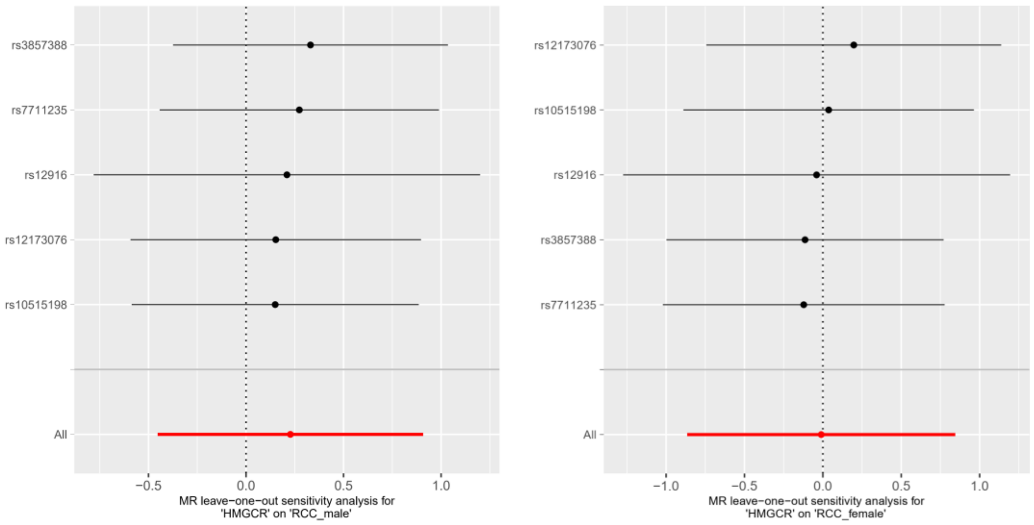

(b) NPC1L1

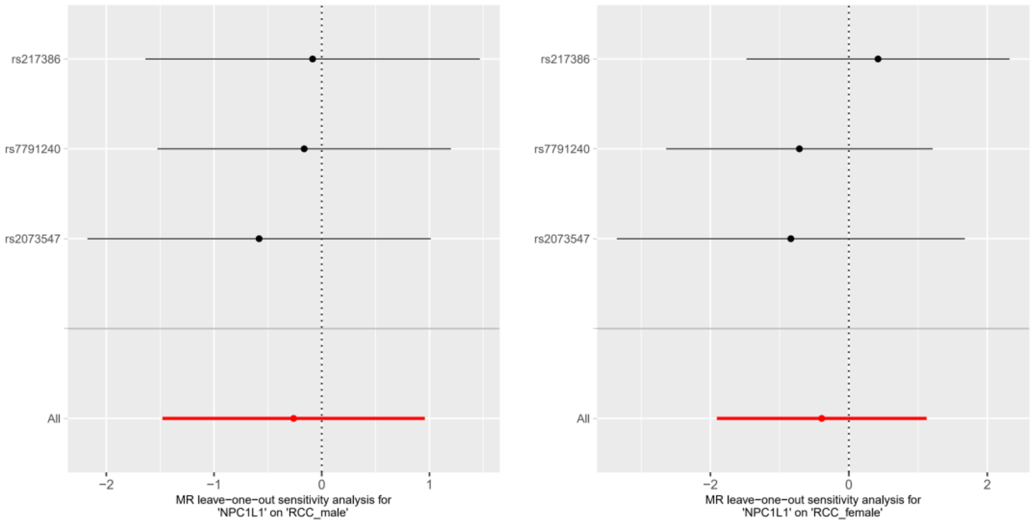

(c) LDLR

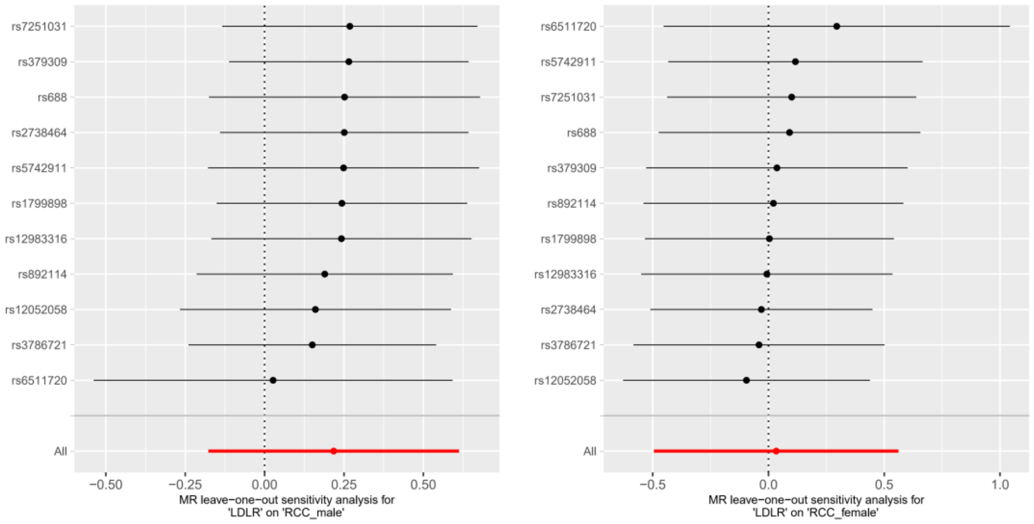

(d) APOB

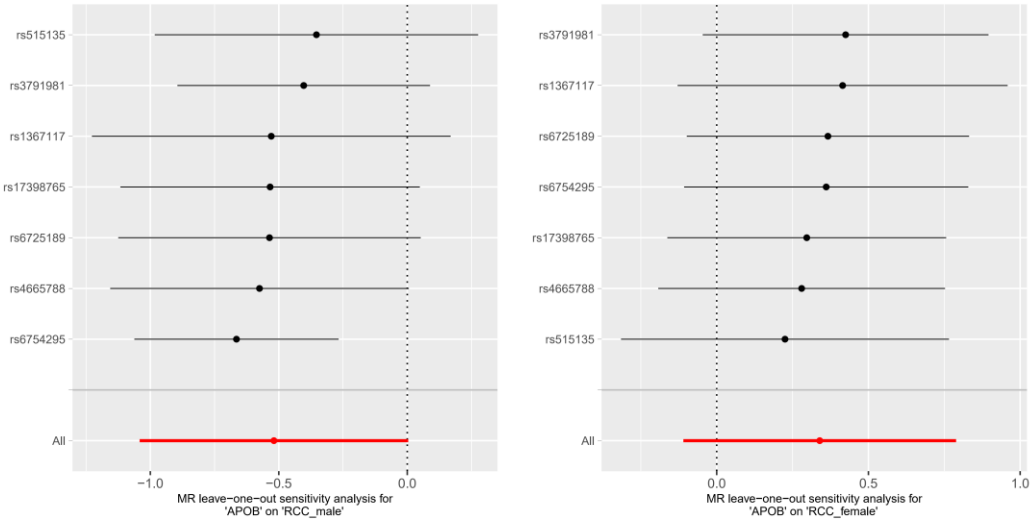

Supplementary Figure 3 **Leave-one-out analysis plots.**

Supplementary Table 13 *HMGCR* variants included in *HMGCR* genetic score and their association with LDL-C in the Global Lipids Genetics Consortium.

SNP	Exposure allele	Exposure allele frequency	LDL Effect Size (mg/dL)	LDL SE	*P*-value	Sample size
rs12916	T	0.5686	-2.3456	0.1216	7.79E-78	168,357
rs17238484	G	0.7467	-2.0064	0.1984	1.35E-21	80,959
rs5909	G	0.8984	-1.9744	0.2816	4.93E-13	89,875
rs2303152	G	0.8799	-1.3536	0.2048	1.04E-09	160,116
rs10066707	G	0.5831	-1.5904	0.1728	2.97E-19	89,888
rs2006760	C	0.8140	-1.7056	0.2432	1.67E-13	89,885

Supplementary Table 14 *NPC1L1* variants included in *NPC1L1* genetic score and their association with LDL-C in the Global Lipids Genetics Consortium.

SNP	Exposure allele	Exposure allele frequency	LDL Effect Size (mg/dL)	LDL SE	*P*-value	Sample size
rs217386	A	0.4077	-1.1253	0.118	1.20E-19	173,021
rs2073547	A	0.8061	-1.5035	0.152	1.92E-21	169,889
rs7791240	T	0.9090	-1.3175	0.202	1.84E-10	161,845
rs10234070	C	0.9037	-0.9145	0.183	1.52E-06	160,051
rs2300414	G	0.9301	-1.0943	0.248	5.45E-06	115,286

Supplementary Table 15 *PCSK9* variants included in *PCSK9* genetic score and their association with LDL-C in the Global Lipids Genetics Consortium.

SNP	Exposure allele	Exposure allele frequency	LDL Effect Size (mg/dL)	LDL SE	*P*-value	Sample size
rs11206510	C	0.1544	-2.6592	0.005	2.38E-53	172,812

rs2479409	A	0.6675	-2.0544	0.0041	2.52E-50	172,970
rs2149041	C	0.8391	-2.0352	0.0049	1.44E-35	172,903
rs2479394	A	0.715	-1.2352	0.0041	1.58E-19	172,953
rs10888897	T	0.3945	-1.6224	0.0042	8.43E-31	165,232
rs7552841	C	0.6346	-1.1776	0.0044	5.40E-15	140,234
rs562556	G	0.1939	-2.048	0.0066	6.16E-21	99,192

Supplementary Table 16 *LDLR* variants included in *LDLR* genetic score and their association with LDL-C in the Global Lipids Genetics Consortium.

SNP	Exposure allele	Exposure allele frequency	LDL Effect Size (mg/dL)	LDL SE	*P*-value	Sample size
rs6511720	T	0.1086	-6.7657	0.1362	3.69E-538	295,826
rs1122608	T	0.2266	-2.1179	0.1075	2.02E-84	262,102
rs688	C	0.5586	-1.728	0.1184	3.04E-48	166,792

Supplementary Table 17 *CETP* variants included in *CETP* genetic score and their association with LDL-C in the Global Lipids Genetics Consortium.

SNP	Exposure allele	Exposure allele frequency	Sample size	LDL Effect Size (mg/dL)	LDL-C (95% CI)	*P*-value
rs3764261	A	0.2942	164,865	-1.690	-1.43, -1.95	2.22E-34
rs1800775	A	0.4802	168,024	-1.318	-1.08, -1.56	8.54E-24
rs1864163	G	0.7322	171,395	-1.398	-1.12, -1.68	7.97E-21
rs9929488	G	0.6966	143,288	-1.194	-0.89, -1.51	8.15E-13
rs9989419	G	0.5950	163,625	-0.886	-0.64, -1.13	2.49E-12
rs12708967	T	0.8034	171,501	-1.120	-0.80, -1.44	3.47E-11
rs289714	A	0.7942	169,344	-1.146	-0.81, -1.49	2.85E-10
rs5880	G	0.9406	161,383	-1.504	-0.90, -2.11	1.59E-06

Supplementary Table 18 Association of Genetically Proxied Inhibition of *HMGCR*, *NPC1L1*, *PCSK9*, *CETP*, *LDLR*, *APOB* with Overall and Sex-specific Renal Cell Carcinoma Risk using instrument variables reported in previous articles.

Targets	Gender	N_{out}	N_{SNPs}	IVW			Weighted median		MR-Egger	
				OR (95% CI)	*P*	*Phet*	OR (95% CI)	*P*	OR (95% CI)	*P*
HMGCR	Male	8143	6	1.004 [0.980-1.030]	0.733	0.81	1.006 [0.985-1.028]	0.588	1.037 [0.893-1.205]	0.630
	Female	5087	6	0.999 [0.968-1.031]	0.935		1.001 [0.974-1.029]	0.934	1.013 [0.874-1.174]	0.860
	Overall	4735	6	1.016 [0.964-1.070]	0.558		1.004 [0.960-1.051]	0.854	0.883 [0.718-1.088]	0.243
NPC1L1	Male	8143	5	0.985 [0.944-1.028]	0.486	0.64	0.983 [0.940-1.027]	0.436	1.091 [0.611-1.947]	0.770
	Female	5087	5	1.006 [0.931-1.087]	0.880		1.007 [0.949-1.070]	0.810	0.882 [0.652-1.193]	0.414
	Overall	4735	5	0.956 [0.882-1.036]	0.271		0.955 [0.884-1.033]	0.253	0.936 [0.529-1.656]	0.821
PCSK9	Male	8143	7	1.022 [1.003-1.043]	**0.026**	0.46	1.027 [1.005-1.049]	0.016	1.032 [0.965-1.104]	0.357
	Female	5087	7	1.010 [0.985-1.034]	0.440		1.001 [0.973-1.029]	0.956	1.018 [0.926-1.120]	0.712
	Overall	4735	7	1.001 [0.965-1.038]	0.973		1.003 [0.962-1.045]	0.884	0.937 [0.772-1.136]	0.508
LDLR	Male	8143	3	1.011 [0.996-1.027]	0.138	0.18	1.013 [0.998-1.029]	0.094	1.015 [0.990-1.041]	0.242
	Female	5087	3	0.994 [0.975-1.013]	0.525		0.993 [0.973-1.013]	0.484	0.986 [0.948-1.026]	0.500
	Overall	4735	3	1.001 [0.972-1.030]	0.965		0.999 [0.971-1.027]	0.935	0.997 [0.872-1.139]	0.962
CETP	Male	8143	8	1.029 [0.995-1.064]	0.098	0.81	1.030 [1.001-1.061]	0.045	1.011 [0.914-1.118]	0.830

Female	5087	8	1.036 [0.993-1.080]	0.102	1.035 [0.999-1.072]	0.056	1.017 [0.851-1.216]	0.849
Overall	4735	7	1.078 [1.008-1.153]	0.029	1.063 [1.005-1.124]	0.033	0.998 [0.809-1.232]	0.987

Abbreviation: IVW, inverse-variance weighted; OR, odds ratio; N_{out}: Number of participants in the renal cell carcinoma genome-wide association study. N_{SNPs}: Numbers of SNPs in each instrument variable. $P_{interaction}$ for heterogeneity between sex-specific effect estimates.

Reference

1 Liu, M., Jiang, Y., Wedow, R., Li, Y., Brazel, D.M., Chen, F., Datta, G., Davila-Velderrain, J., McGuire, D., Tian, C. *et al.* (2019) Association studies of up to 1.2 million individuals yield new insights into the genetic etiology of tobacco and alcohol use. *Nature genetics*, **51**, 237-244.

2 Wootton, R.E., Richmond, R.C., Stuijfzand, B.G., Lawn, R.B., Sallis, H.M., Taylor, G.M.J., Hemani, G., Jones, H.J., Zammit, S., Davey Smith, G. *et al.* (2020) Evidence for causal effects of lifetime smoking on risk for depression and schizophrenia: a Mendelian randomisation study. *Psychological medicine*, **50**, 2435-2443.

3 Yengo, L., Sidorenko, J., Kemper, K.E., Zheng, Z., Wood, A.R., Weedon, M.N., Frayling, T.M., Hirschhorn, J., Yang, J. and Visscher, P.M. (2018) Meta-analysis of genome-wide association studies for height and body mass index in ∼700000 individuals of European ancestry. *Human molecular genetics*, **27**, 3641-3649.

4 Pulit, S.L., Stoneman, C., Morris, A.P., Wood, A.R., Glastonbury, C.A., Tyrrell, J., Yengo, L., Ferreira, T., Marouli, E., Ji, Y. *et al.* (2019) Meta-analysis of genome-wide association studies for body fat distribution in 694 649 individuals of European ancestry. *Human molecular genetics*, **28**, 166-174.

5 Evangelou, E., Warren, H.R., Mosen-Ansorena, D., Mifsud, B., Pazoki, R., Gao, H., Ntritsos, G., Dimou, N., Cabrera, C.P., Karaman, I. *et al.* (2018) Genetic analysis of over 1 million people identifies 535 new loci associated with blood pressure traits. *Nature genetics*, **50**, 1412-1425.

6 Dupuis, J., Langenberg, C., Prokopenko, I., Saxena, R., Soranzo, N., Jackson, A.U., Wheeler, E., Glazer, N.L., Bouatia-Naji, N., Gloyn, A.L. *et al.* (2010) New genetic loci implicated in fasting glucose homeostasis and their impact on type 2 diabetes risk. *Nature genetics*, **42**, 105-116.

7 Morris, A.P., Voight, B.F., Teslovich, T.M., Ferreira, T., Segrè, A.V., Steinthorsdottir, V., Strawbridge, R.J., Khan, H., Grallert, H., Mahajan, A. *et al.* (2012) Large-scale association analysis provides insights into the genetic architecture and pathophysiology of type 2 diabetes. *Nature genetics*, **44**, 981-

990.

8 Gaulton, K.J., Ferreira, T., Lee, Y., Raimondo, A., Mägi, R., Reschen, M.E., Mahajan, A., Locke, A., Rayner, N.W., Robertson, N. *et al.* (2015) Genetic fine mapping and genomic annotation defines causal mechanisms at type 2 diabetes susceptibility loci. *Nature genetics*, **47**, 1415-1425.

9 Pattaro, C., Teumer, A., Gorski, M., Chu, A.Y., Li, M., Mijatovic, V., Garnaas, M., Tin, A., Sorice, R., Li, Y. *et al.* (2016) Genetic associations at 53 loci highlight cell types and biological pathways relevant for kidney function. *Nature communications*, **7**, 10023.

10 Rees, J.M.B., Wood, A.M. and Burgess, S. (2017) Extending the MR-Egger method for multivariable Mendelian randomization to correct for both measured and unmeasured pleiotropy. *Statistics in medicine*, **36**, 4705-4718.

11 Burgess, S. and Thompson, S.G. (2015) Multivariable Mendelian randomization: the use of pleiotropic genetic variants to estimate causal effects. *American journal of epidemiology*, **181**, 251-260.

12 Ference, B.A., Majeed, F., Penumetcha, R., Flack, J.M. and Brook, R.D. (2015) Effect of naturally random allocation to lower low-density lipoprotein cholesterol on the risk of coronary heart disease mediated by polymorphisms in NPC1L1, HMGCR, or both: a 2 × 2 factorial Mendelian randomization study. *Journal of the American College of Cardiology*, **65**, 1552-1561.

13 Ference, B.A., Robinson, J.G., Brook, R.D., Catapano, A.L., Chapman, M.J., Neff, D.R., Voros, S., Giugliano, R.P., Davey Smith, G., Fazio, S. *et al.* (2016) Variation in PCSK9 and HMGCR and Risk of Cardiovascular Disease and Diabetes. *The New England journal of medicine*, **375**, 2144-2153.

14 Ference, B.A., Kastelein, J.J.P., Ginsberg, H.N., Chapman, M.J., Nicholls, S.J., Ray, K.K., Packard, C.J., Laufs, U., Brook, R.D., Oliver-Williams, C. *et al.* (2017) Association of Genetic Variants Related to CETP Inhibitors and Statins With Lipoprotein Levels and Cardiovascular Risk. *Jama*, **318**, 947-956.

15 Williams, D.M., Finan, C., Schmidt, A.F., Burgess, S. and Hingorani, A.D. (2020) Lipid lowering and Alzheimer disease risk: A mendelian randomization

study. *Ann Neurol*, **87**, 30-39.
